# Robust morphogenesis by chaotic dynamics

**DOI:** 10.1038/s41598-023-34041-x

**Published:** 2023-05-09

**Authors:** J. Reinitz, S. Vakulenko, I. Sudakow, D. Grigoriev

**Affiliations:** 1 Departments of Statistics, Ecology and Evolution, Molecular Genetics and Cell Biology, University of Chicago, Chicago, IL 60637 USA; 2 Institute for Problems in Mechanical Engineering, Russian Academy of Sciences, Saint Petersburg, 199178 Russia; 3 Saint Petersburg Electrotechnical University, Saint Petersburg, 197022 Russia; 4 School of Mathematics and Statistics, The Open University, Milton Keynes, MK7 6AA, UK; 5 CNRS, Mathématiques, Université de Lille, Villeneuve d’Ascq, 59655 France

**Keywords:** Morphogenesis, Applied mathematics

## Abstract

This research illustrates that complex dynamics of gene products enable the creation of any prescribed cellular differentiation patterns. These complex dynamics can take the form of chaotic, stochastic, or noisy chaotic dynamics. Based on this outcome and previous research, it is established that a generic open chemical reactor can generate an exceptionally large number of different cellular patterns. The mechanism of pattern generation is robust under perturbations and it is based on a combination of Turing’s machines, Turing instability and L. Wolpert’s gradients. These results can help us to explain the formidable adaptive capacities of biochemical systems.

## Introduction

More than 50 years ago, Lewis Wolpert^1^ proposed the positional information (PI) model to describe patterns of different cell types. Seventy years ago, Alan Turing introduced the idea of patterns originating from homogeneous states by reaction-diffusion mechanism (RDM)^2^. In both conceptual models, an organism is represented as a pattern consisting of different cells. Both Turing’s and Wolpert’s approaches assume that morphogens, special reagents, can change cell states. Modern research has shown that the French flag and reaction-diffusion models do not capture all details of real biological patterning systems^3^, and the relationship between them remains unclear although some ideas are proposed by Green and Sharpe^4^.

In this paper, inspired by the ideas of Brenner^5^ and Jacob about evolutionary tinkering^6^, we show that a combination of RDM and PI mechanism gives a universal robust pattern mechanism. Brenner emphasized the importance of Turing’s ideas in the context of his famous machine. A Turing machine is a mathematical model of computation that defines an abstract machine that manipulates symbols on a tape according to some rules. The machine stops when it arrives at a prescribed terminal state. In its turn, Jacob introduced the concept of tinkering (evolution uses all available means^6^).

**Figure 1:**
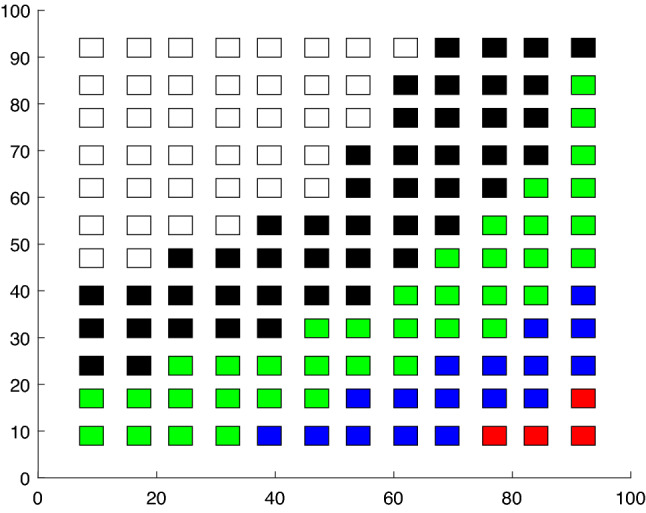
This picture illustrates the concept of a developmental program. On the x-axis, there is a one-dimensional "organism" composed of various cells that form layered patterns at time moments $t_0, t_1, ..., t_n$. Different types of cells are shown in different colors. The top row of the cells emerges at the last time moment $t_n$, the previous row appears at $t=t_{n-1}$ and the bottom row arises at the initial time moment $t=t_0$. Each column shows how the corresponding cell (located at the bottom) changes its type in time. We refer to this two-dimensional spatiotemporal pattern consisting of cells of different discrete types as a cellular developmental program (CDP).

We show the dynamics of a generic open chemical reactor is capable to generate an exponentially large number of different attractors, which may be chaotic, periodic, or steady states. The chaotic or periodic attractors can occur if our reaction-diffusion model is not gradient-like and not monotone. The results hold under certain mathematical conditions, which admit a transparent chemical interpretation for two-component systems: one of the reagents is neither an activator nor an inhibitor for the other reagent, reagent diffusion coefficients are sharply different and the space dimension of the system $\ge 2$. The second result is that such open chemical systems can be used as a sort of Turing machine (TM), which can produce an exponentially large number of different cellular differentiation programs (CDPs, see Fig. 1). CDP is a program, which, by morphogene concentrations, produces cell patterns unfolding in time and space, for example, gastrulation patterns, or somitogenesis periodical structures^7,8^. The number of possible CDPs increases exponentially in the system size.

The proposed cell pattern formation mechanism is robust against fluctuations and perturbations. So, a combination of two of Turing’s basic ideas (machines and instability in chemical systems) leads to general results, which sharply extend the Turing instability^2^ model. The combined Turing-Wolpert model makes it possible to obtain evolving over time patterns that are much more complex than zebra stripes.

**Figure 2:**
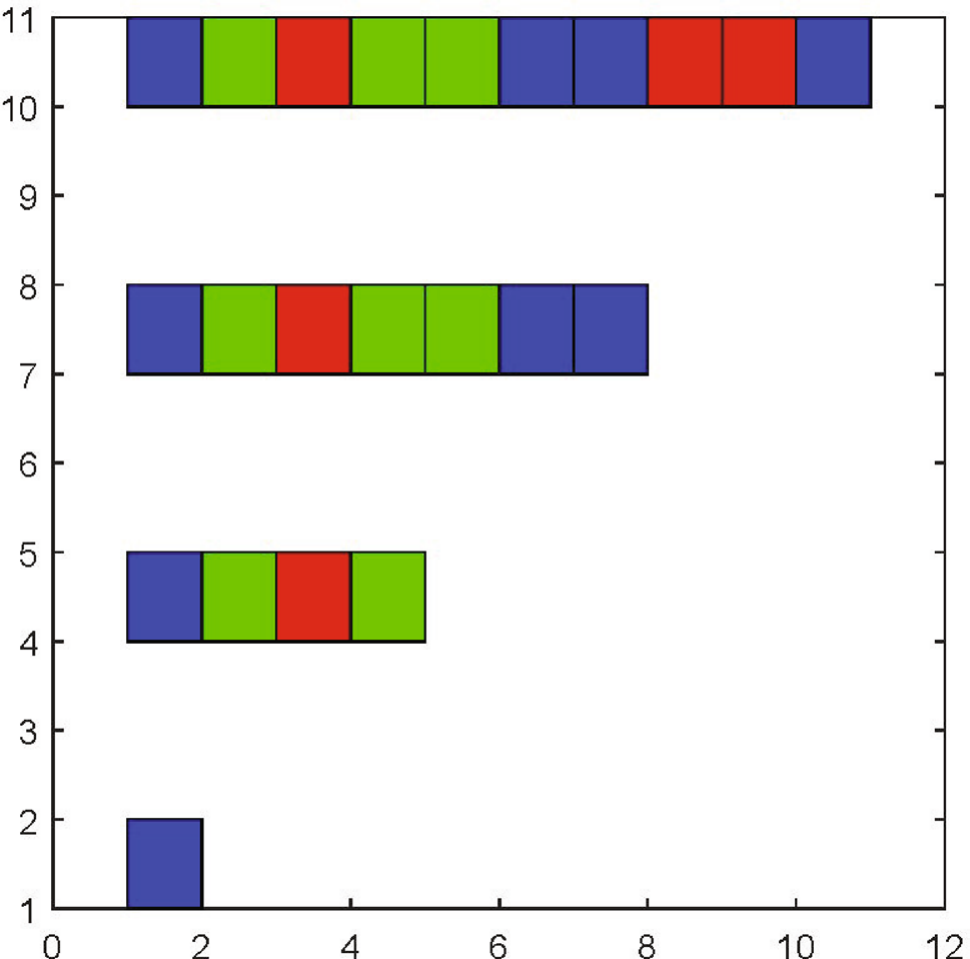
This image shows the generalized French Flag model. A cell pattern can be considered as a string in the alphabet (red, blue, or green). This pattern can be produced by chaotic dynamics as follows. Let *u* be a vector of morphogen’s concentrations, which lies in morphogen concentration space ${{\mathscr {U}}}$. Suppose that this space is split into three subdomains ${{\mathscr {U}}}_j$, $j=1,2,3$. If $u \in {{\mathscr {U}}}_1$, morphogen concentrations induce differentiation into a red cell, and if $u \in {\mathscr U}_2$, $u \in {{\mathscr {U}}}_3$ then one has blue and green cells, respectively. When *u*-dynamics is governed by a chaotic (noisy) system, there exist time moments $t_1, t_2, ..., t_m$ such that the *u* states enter for ${{\mathscr {U}}}_j$ at certain time moments: there exist $t, t+\Delta T, ..., t+m \Delta T$ such that state $u(t+j\Delta t)$ lies in the corresponding domain ${\mathscr U}_{a_j}$. In the considered case, we have a string $\textbf{a}=2313322112$. It is important that the choice of chaotic dynamics and specific choice of partition ${{\mathscr {U}}}_j$ is not essential. Any stochastic (chaotic) ergodic dynamics is capable, earlier or later, to generate the needed string.

The mechanism of attractor and pattern generation can be sketched as follows. One can show (see “Methods”, and the work^9^) that a generic open chemical system is capable to generate a number of chaotic or periodic attractors if it involves appropriate spatial heterogeneities. These inhomogeneities work as activators and excite the formation of sharply localized segments like to Drosophila segmentation. Segments correspond to local maxima of slow reagent concentration. These segments interact in a long-range manner via the fast diffusing component, all these mechanisms remind ones described in the manuscript^10^ but we are capable to prove that they can lead to chaos. The chaotic dynamics allows us to generate complicated cell patterns in space and in time that can be shown by the results from these works^11–14^ (see Fig. 2).

According to Meinhardt^10^, to generate complex patterns, a reaction-diffusion mechanism should include local self-activation and long-range inhibition. The new mechanism, proposed in this paper, which is capable to create a number of complicated local attractors, is more sophisticated. It also uses a local activation leading to layered patterns for the slow diffusing component but instead of long-range inhibition, we have a more complicated interaction structure. In some space domains, the slow reagent serves as an activator while in other ones it works as an inhibitor. Such a mosaic (mixed) structure of activation and inhibition arises due to spatial gradients. A well-adjusted spatial gradient causes the formation of many well-localized segments, where the slowly diffusing reagent *u* is concentrated. The reagent *u* in these local layers interacts with fast diffusing component *v*, which, in turn, acts on those layers and so we obtain nonlinear non-local feedback and complex dynamics. Due to the mosaic structure of activation and inhibition, this mechanism can lead to chaos. Dynamics induced by each group of interacting segments generate different local attractors.

Biological systems are affected by perturbations (including stochastic ones) and the concentrations of gene products fluctuate. Moreover, pattern formation goes under environmental noises and mutations. Nonetheless, we know that the final result of pattern formation is remarkably stable, i.e., the patterning of real organisms is a quite robust (canalized) process. Starting with seminal works^15,16^ a number of works were focused on the canalization and robustness problem, see, for example, the research^17–20^ and the review^21^.

By Turing machine (TM) theory, we show that robustness can be provided if the patterning goes into some stages (a multistage process) and for each stage, there is a stop signal, which informs that an intermediate targeted pattern is achieved. Therefore, the Turing machine theory helps to show the formidable ability of biochemical systems to generate different structures and the robustness of this generation.

Note that the pattern and chaos emergence process can be considered as self-organization, and therefore, the Turing machines arise as a result of self-organization. The question of the computation power of self-organization in physical systems remains highly controversial: some authors believe^22^ that it can make hypercomputing (something stronger than Turing machines). This complex question is outside of our scope in this research.

Moreover, we conducted a theoretical analysis and performed simulations using white noise, prioritizing simplicity and utilizing the classical mathematical theory primarily developed for white noise and Wiener processes (see the study^23^ for an excellent statement). In this work, we do not pretend to describe bifurcations between different attractors induced by noises because our method uses structural stability under deterministic and random perturbations^24,25^.

## Results

### Model

Let us consider the following reaction-diffusion model with spatial gradients

1 $$\begin{aligned} u_t =\textbf{D} \Delta u + f(u) + \eta , \end{aligned}$$

where $u=(u_1(x,y,t), u_2(x,y,t), \ldots , u_m(x,y,t))$ are unknown functions of reagent concentrations defined on $\Omega \times \{ t \ge 0 \}$, $\eta (x,y)=(\eta _1, \ldots , \eta _m)$ is a vector-valued function, which can be considered as a spatially heterogeneous source (possibly involving a small space-temporal noisy term), $\Omega$ is the strip $(-\infty , \infty ) \times (0,1) \subset \mathbf{R^2}$, and $\textbf{D}=diag(d_1, \ldots , d_m)$ is a diagonal matrix of diffusion coefficients $d_i >0$. We assume that reaction terms $f_i(u)$ are smooth, for example, polynomials. We complement (Eq. 1) by standard initial and boundary conditions, for example, at $y=0,1$ for certain $u_i$ we set the zero Neumann boundary conditions and for other ones, we set the zero Dirichlet ones. To simplify the problem, we set periodic boundary conditions along *x* assuming that all the functions $u_i^0$ that define initial data are $2\pi$-periodic in *x*. The initial boundary value problem (IBVP) is defined by Eq. (1), and standard boundary and initial conditions define a family of local semiflows $S^t_\textbf{P}$. Each semiflow $S^t_\textbf{P}$ depends on the problem parameters $\textbf{P}$, which are external gradients $\eta (x,y)$ and diffusion coefficients $d_i$^9^. As a simple example, one can consider the following two-component system:

2 $$\begin{aligned} \frac{\partial u}{\partial t}&=d_u \Delta u -\lambda _1 u + f(u,{v}) + \zeta , \end{aligned}$$

3 $$\begin{aligned} \frac{\partial v}{\partial t}&=D_v \Delta v -\lambda _2 v + {g}(u, {v}) + {\eta }, \end{aligned}$$

where $\eta (x,y), \zeta (x,y)$ are smooth functions, *f*, *g* are smooth nonlinearities, for example, polynomials, (*x*, *y*) lies in the strip $[-\infty , \infty ] \times [0, L_2]$. For mathematical tractability, we consider periodic (or zero Neumann) boundary conditions $u(x+L_1,y, t)=u(x, y), v(x+L_1,y, t)=v(x, y, t)$, the zero Neumann condition for *u* at the top and the bottom $y=0, L_2$ and and the zero Dirichlet conditions for *v* at $y=0, L_2$. We also set initial conditions for *u*, *v*. We consider degradation coefficients $\lambda _i \ge 0$, diffusion coefficient $d_u, D_v$ and sources $\eta , \zeta$ (which can be interpreted as maternal morphogen gradients) as a parameter $\textbf{P}$.

### Main results

The following assertions stated in a non-formal manner, unwrap the main results. The first one (Claim I) is a corollary of previous results^9^:

*For generic* *f*, *g* *open system* (Eqs. 2, 3) *enjoys the property of Universal Dynamical Approximation (UDA): these systems can generate all possible finite dimensional structurally stable dynamics when we vary their parameters* **P** *. In particular, these systems are capable to generate hyperbolic chaotic dynamics, such as Smale horseshoe, Anosov flow, etc. These chaotic sets can be at any dimension.*

You can find more details and formal definitions of UDA in the upcoming section on the methods. Note that the reaction part is fixed and to obtain the needed dynamics, we adjust the diffusion, degradation coefficients and external gradients $\zeta , \eta$. The words ‘generic open’ means the choice of *f*, *g* excludes gradient-like and monotone reaction-diffusion systems. In fact, gradient-like systems have the Lyapunov functions which can be interpreted physically as energy decreasing along trajectories. In general, the existence of such energy leads to convergence to equilibria and rules out stable chaotic or periodic regimes (although an unstable complicated large-time behavior is possible^26^). In our case the sense of ’genericity’ (the fundamental concept introduced by Thom^27^) is that if some reaction parts *f*, *g* are ’bad’, i.e., our assertion does not hold, then a small smooth correction to *f*, *g* transforms these functions into ’good’ ones and for them the assertion is valid. Note that the conditions on *f*, *g* can be formulated explicitly^9^, in particular, it can be checked that these conditions hold for the Brusselator and other models.

For general multicomponent systems, one can show the following (Claim II):

*Under some conditions on the reaction part* *f*, *the systems defined by* Eq. (1) *are capable to generate* $2^{M_a}$ *different structurally stable local attractors (which may be periodic or even chaotic), where* $M_a > C_f m d_{min}^{-1/4}$, $d_{min}$ *denotes the minimal diffusion coefficient,* $C_f>0$ *is a constant depending on the nonlinear part* *f*, *m* *the number of components (involved reagents). *

We can consider external sources $\eta (x,y)$ in Eqs. (2) and (3) as spatial concentrations of certain maternal morphogens (for example, Bicoid and Nanos in Drosophila^28^). To obtain a complicated attractor, we need an appropriate choice of functions $\eta (x,y)$. We can thus consider those functions as carrying positional information, which helps to create a spatiotemporal pattern. Moreover, we note that in order to have a complex pattern *u*(*x*, *y*, *t*) we need diffusion coefficients of different orders, i.e., some reagents should diffuse much faster than others. So, our result means that under certain conditions on diffusion coefficients and on the reaction part the positional information can be transformed into a spatiotemporal structure with complex large-time behavior.

In the section on the methods, we show (following the study^13^) how chaotic attractors can serve as cellular developmental programs (CDPs) and create complicated spatiotemporal cell patterns. Therefore, it is shown that dynamical systems are given by Eq. (1) (which realize combined Truing-Wolpert mechanism) are capable to realize all possible cell developmental programs (CDPs). The maximal possible number of these programs is not less than $2^{M_{dvp}}$ with $M_{dvp} \propto M_a$. So, we can formulate the following non-formal assertion:

**Assertion on the superpower of Turing–Wolpert mechanism**

*Combined Turing–Wolpert mechanism can produce any cell patterns unfolding in space and time and has a formidable capacity: it is capable to produce exponentially many different cell patterns.*

Note that CDPs exhibit more complicated cell dynamics than cellular automata with local interaction. In fact, in the CDP case two subsequent in-time rows of cells can be arbitrary (see Fig. 1) whereas if they are generated by a cellular automaton with a local interaction, there should be correlations between subsequent rows.

The proof of Claim I can be obtained by results in the paper^9^ and by a simple construction from dynamical systems theory. The proof of Claim II is based on Claim I. Below, we outline the main ideas of these demonstrations. Moreover, in Supplementary Material (SM), we consider two-component reaction-diffusion systems with spatial inhomogeneities, which can simulate the known Rössler system exhibiting chaotic behavior. This example and simulations made for its finite difference analog illustrate the main principles of attractor and pattern generation stated below, in particular, the complex spatial structure of activation and inhibition by a slow diffusing component, and the formation of the layers (segments).

## Methods

### Stochastic Turing machine and cell pattern generation

Let us draw key points of our approach. Really existing organisms can include tens and hundreds of types of differentiated cells. The most effective scheme to handle this variety of patterns is to use a binary tree for cell differentiation. As an example, let us consider an organism consisting of 4 cell types (generalization on larger sets of cell types can be done in a straightforward way). We denote these cell types by *gr*, *wr*, *gb*, and *wb*. This notation can be explained as follows. Suppose cells *r* and *b* arise from non-differentiated cells acquiring features *r* and *b*. Further, at the second stage of differentiation, a differentiated cell can acquire additional features *g* and *w*. We encode these cells *gr*, *wr*, *gb* and *wb* as 00, 01, 10 and 11, respectively. For example, the string $a=(00, 01, 01, 01, 11, 10, 00, 11, 11)$ corresponds to cell pattern *gr*, *wr*, *wr*, *wr*, *wb*, *gb*, *gr*, *wb*, *wb*. Further, we consider, for simplicity, binary strings consisting of *r*, *b* cells.

#### Dynamical systems and cell patterns

Consider a layered one-dimensional (1*D*) pattern consisting of cells of two types, red and blue. We decompose our ’organism’ in small domains of equal length occupied by cells. Then the pattern of red and blue cells can be considered as a string, for example, a periodic string (*rbrbrbrb*) (case **P**), a simple string (*rrrrbbb*) (case **S**), or a general non-periodic one (case **G**). Suppose the cell type depends on a morphogen $u \in (0,1)$ level, say, for $u \in R_1=(0, 1/2)$ we have the *r*-cell, and for $u \in R_2=(1/2, 1)$ we have the *b*-cell. Then the periodic pattern **G** can be obtained by a periodical morphogen concentration via the Turing instability, and the simple pattern **S** can be generated by a monotone morphogen gradient via Wolpert’s mechanism. However, to obtain a complex non-periodic pattern of a large length we need sophisticated concentration morphogene profiles *u*(*x*). It is hard to obtain such *u*-gradients by diffusion from a source. Moreover, we would like to generate any prescribed patterns by a universal and short model. In fact, we know that many genes making morphogenesis are shared between many different organisms. These genes are highly conservative in evolution^29,30^. On the other hand, we also know that universal TMs are capable to generate any strings in time and they admit a compact description. Moreover, any time strings can be obtained by chaotic dynamical systems^11,12^, finite automata and Markov chains (see below). So, there are simple dynamic models simulating TMs and generating all possible time cell strings for a few of cell types.

We show how to use stochastic models to generate prescribed time strings. These models have a sufficient number of states (to simulate all types of cells) and function in time up to a stop signal. The stop signal mechanism, borrowed from TM theory, provides canalization (robustness) and regeneration.

Suppose that we would like to obtain a phenotype given a pattern $u(x,s^*)$ and encoded by a target gene expression string $s^*=s_1^* \cdots s_{m}^*$, where $s_i \in S^m=\{0,1\}^m$. At each time moment *t* we have $n=2^{m}$ states $s \in \{0,1\}^{m}$. Let $w(s, s^{\prime }, \xi )$ be the transition probability from $s^{\prime }$ to *s* per one step depending on the environment state $\xi$. Then stochastic gene expression dynamics can be defined, for example, by the following Markov chain

4 $$\begin{aligned} p_s(t+\Delta t)= \sum _{s^{\prime } \in S^m} w(s, s^{\prime }, \xi (t)) p_{s^{\prime }}(t) \end{aligned}$$

where $p_s(t)$ denotes the probability to be in the state *s* at the moment *t*. Assume that the terminal state is absorbing, i.e. the process stops when $s=s^*$ (i.e. $w(s, s^*)=0$ for all $s^*$). This dynamical model includes noisy and chaotic dynamical systems. The main idea is that under fairly general conditions we reach the target state $s^*$ within a bounded time period $\tau (s^*)$. This time is a random quantity, however, for many processes $\tau (s^*)$ can be estimated. This construction describes a realization of a stochastic Turing machine, or more specifically, a random finite automaton, where $s^*$ is a terminal state. Note that dependence on the environment parameter $\xi$ can be important for plant development, where morphogenesis depends on environmental cues. The morphogenesis time $\tau$ is proportional to the number of steps in time to reach the target terminal $s^*$ state.

This stochastic mechanism is feasible, however, only when the state number *n* is not too large. We estimated the dependence $\tau$ on the state number *n* by numerical simulations for the Markov chains with constant transition probabilities. It is obtained that the average $E\tau _{\epsilon }$, where the expected value is taken over all absorbing Markov chains, is a linear function of the state number *n*. The standard deviation of $\tau _{\epsilon }$ is small for large *n* with respect to $E\tau _{\epsilon }$ (see Figs. A7, A8 and A9 in SM).

So, for large *n* we have a large $\tau _{\epsilon }(n) \approx const \ n$ and for too large *n* the stochastic morphogenesis may continue for a long time. We can overcome this difficulty assuming that the long target pattern $s_1^* \cdots s_n^*$ is split into intermediate target patterns $s^{*, k}=s_{m_k}^* \cdots s_{m_{k+1}}^*$, where the long string $s^*$ is a concatenation of shorter strings $s^{*, k}$, $k=1, \dots , p$. The index *k* indicates the *k*-th stage of morphogenesis.

Then the full time $\tau _{\epsilon }(s^*)$ needed to obtain the target string is the sum $\tau (s^*)=\sum _{k=1}^{N_m} \tau (s^{*, k})$. If all the processes are independent, and a new process starts just the previous stage is completed, then for large numbers $N_m$ the total time $\tau (s^*)$ will be very stable due to classical theorems of probability theory (although the times for separate stages are random and can fluctuate). So, we obtain that the stochastic multistage morphogenesis is a well-canalized process, which is robust against external perturbations $\xi$.

Let us consider a simple example: a random search of a target string on *m*-dimensional Boolean hypercube $S^m$. Let $s^*$ be a target pattern, which is uniformly randomly chosen as a vertex of $S^m$. We choose another random vertex $s^0$ as a starting state. At each step, we move from the current vertex to a neighboring one (that corresponds to a flip of some component $s_j$). Our walk ends when we reach the target state. It is clear that for a random initial state, such a search continues $\tau =O(2^{m/2})$ steps (see Fig. A8, the top right line in SM). Note that the random walks on hypercube are considered in the research^31^.

However, if the process goes in a few stages then the time $\tau$ is essentially smaller and the fluctuations of $\tau$ are smaller. In fact, let us represent a target string $s^*$ as a concatenation of several strings $s^{*,1}, ... , s^{*, k}$. Suppose for simplicity that lengths $l(s^{*, j})$ (of $s^{*, j}$ are equal: $l(s^{*, j})=m^{\prime }= m/k$ and the search of $s^{*}$ goes in a *k* stages. First we go to $s^{*,1}$ by a random walk on the hypercube $S^{m^{\prime }}$ of dimension $m^{\prime }$. Just the process reaches $s^{*,1}$ we fix components of $m^{\prime }=m/k$ of the first components of *s*. Then we move to $s^{*,2}$ by a random walk on the second hypercube of dimension $m^{\prime }$, etc. All this search requires $O( k 2^{m^{\prime }})$ steps. This means that we have an exponential acceleration by a decomposition (the results of numerical simulations are shown in Fig. A8 in SM). We define the normalized standard deviation $\sigma (\tau )$ of $\tau$ as $\sigma (\tau )=E\tau /\sqrt{Var \ \tau }$, where *EX* and $Var \ X$ denotes the expected value and the variation of random quantity *X*. Then computations show that $\sigma (\tau ) \approx 1/\sqrt{k}$ and $\sigma (\tau ) \approx 1$ when the process goes in a single stage. A stochastic dynamics defined by Eq. (4) can be used to make morphogenesis. The key idea is as follows. Each pattern is encoded by a genotype *s*. We suppose that the pattern corresponding to a viable organism (obtained by an evolution) is encoded by $s^*$. Then we use $s^*$ as a terminal (absorbing) state in the dynamics defined by the Master equation (4). In the next subsection, we consider the realization of dynamics (Eq. 4) and TMs via reaction-diffusion models.

### Universal dynamical approximation

Let us outline the concept of Universal Dynamical Approximation (UDA) (that term is inspired by the work^32^). To explain this concept, remind that many neural networks such as multilayered perceptrons (MLP) enjoy the property of universal approximation: for each prescribed sufficiently smooth output function *f* defined on a compact domain $D \subset \textbf{R}^n$ of a Euclidean space $\textbf{R}^n$ and each $\epsilon >0$ we can adjust parameters of MLP in such a way that the output of $f_{net}(q)$ of the network is $\epsilon$-close to *f*(*q*) for all entries *q* from the domain *D*^33^.

The UDA concept generalizes universal approximation for the networks. Many evolution equations under reasonable boundary conditions define global semiflows in the appropriate functional phase spaces^34,35^. We denote such semiflows by $S^t_\textbf{P}$ and they depend on the parameters $\textbf{P}$ involved in equations, and boundary conditions. More formally, let us consider an evolution equation in a Banach space $\textbf{B}$ depending on the parameter $\textbf{P}$:

5 $$\begin{aligned} u_t= \textbf{A} u + F(u, \textbf{P}). \end{aligned}$$

Assume that for some $\textbf{P}$ this equation generates a global semiflow $S^t$. We obtain then a family ${\mathscr {F}}$ of global semiflows $S^t_\textbf{P}$ where each semiflow depends on the parameter $\textbf{P}$.

Suppose for an integer $n> 0$ there is an appropriate value $\textbf{P}_n$ of the parameter $\textbf{P}$ such that the corresponding global semiflow $S^t_{\textbf{P}_n}$ has an *n*-dimensional normally hyperbolic locally invariant manifold ${{\mathscr {M}}}_n$ embedded in our phase space $\textbf{B}$ by a $C^1$-smooth map defined on a ball $\textbf{B}^n \subset \textbf{R}^n$.

The restriction of semiflow $S^t_{\textbf{P}_n}$ to ${{\mathscr {M}}}_n$ is defined then by a vector field *Q* on ${{\mathscr {M}}}^n$. Then we say that the family $S^t_\textbf{P}$ realizes the vector field *Q* (this terminology is coined by Poláčik^26,36^).

The family $S^t_\textbf{P}$ enjoys UDA if for each dimension *n* this family realizes a dense (in the norm $C^1(\textbf{B}^n)$) set of vector fields *Q* on the ball $\textbf{B}^n$.

#### Corollary 1

*If the family* $S^t_\textbf{P}$ *has UDA property, the Theorem on Persistence of Hyperbolic Sets implies that some semiflows* $S^t_\textbf{P}$ *exhibit a chaotic large-time behavior*.

In other words, one can say that the UDA semiflows can simulate, by parameter variations, any finite-dimensional dynamics defined by a system of ordinary differential equations on the domain $D \subset \textbf{R}^n$ within any prescribed accuracy $\epsilon$ (in $C^1(D)$-norm). This property implies that semiflows $S_\textbf{P}^t$ can generate all structurally stable dynamics (up to orbital topological equivalency). Among the systems enjoying UDA, there are a number of fundamental ones: quasilinear parabolic equations^26,36^, time-continuous and time-recurrent neural networks^37^, a large class of reaction-diffusion systems with heterogeneous sources^9^, generalized Lotka-Volterra system^38^, Oberbeck-Boussinesq model^39^. Also, the Euler equations on multidimensional manifolds exhibit similar properties^32^. Note that for time continuous and time recurrent neural networks and generalized Lotka-Volterra system the UDA property follows from Universal Approximation Theorem for MLP^37^. Such systems could be Turing complete and used as TMs.

### Attractor and pattern formation mechanisms

We follow the work^9^ and for simplicity let us restrict ourselves by two-component case $m=2$ (see Eqs. 2 and 3). Let the corresponding diffusion coefficients be sharply different: $d_1=d_u=\epsilon ^2<< d_2=D_v$, where $\epsilon >0$ is a small parameter. The main idea is as follows. We use local excitation for slow component *u* and long-range non-local interaction between *v* and *u*. First spatial gradients $\eta , \zeta$ cause the formation of many well-localized segments, where the slowly diffusing reagent *u* is concentrated.

This reagent concentration *u* interacts with fast diffusing component *v*, which, in turn, acts on *u* and so we obtain nonlinear non-local feedback and complex dynamics. Dynamics induced by each group of interacting segments generate different local attractors. We can have at least $N_{loc}$ of such groups. So, by adjusting different initial data for *u*, *v* we can obtain $2^{N_{loc}}$ of different local attractors, and each attractor (if it is chaotic and structurally stable) can generate the corresponding CDPs. So, we show that fairly general chemical reactors with spatial gradients can create chemical patterns consisting of many narrow segments and those patterns help to activate a number of different CDPs.

In more detail, for each $N =O({\epsilon }^{-1/2})$ we can adjust such gradients $\eta , \zeta$ that the *u*-component is a linear combination of *N* fixed smooth functions $\psi _i(x)$ with coefficients $X_i(t)$ slowly depending on time. Each $\psi _i$ is well localized at a point ${\bar{x}}_i$: it is an exponentially decreasing function of $\epsilon ^{-1}(x-{\bar{x}}_i)$. Then the reagent *u* is concentrated at the right lines $x={\bar{x}}_i$ and the expression level of *u* at *i*-th line is determined by the magnitude $X_i$. Thus we are dealing with an analog of the segmentation process omnipresent in morphogenesis (in fact, it reminds segmentation in Drosophila, where the morphogen Bicoid gradient causes the formation of segments as a result of a complex interaction with other proteins^40–42^). The large time dynamics of the magnitudes $X_i$ are defined by a big *N*-component coupled oscillator system. The *i*-th oscillator with the state $X_i(t)$ interacts with other ones via linear interaction terms. Due to localization properties of $\psi _i$ if the distances between oscillators are much more than $\epsilon$ this interaction force between oscillators is exponentially weak. Using this interaction fading property we can decompose the coupled oscillator system into $M_a$ almost independent dynamical systems, where each system involves ${\bar{n}}=N/M_a$ variables. For this end, we decompose the points ${\bar{x}}_i$, $i=1, ..., N$ into groups $\bar{x}_{j, k}$, where $k=1, ..., M_a$ and $j=1, ..., {\bar{n}}$. The points of the *k*-th group are located close to each other (distances of the order $\epsilon ^{1/2}$) while segments belonging to different groups are separated (distances between segments from different groups are $> 1/2N$). By a variation of the system (Eq. 1) parameters, one can obtain the following. The *k*-th segment group generates an *n*-dimensional dynamical system, which has at least two local hyperbolic attractors ${{\mathscr {A}}}_k^{(l)}$, $l=1,2$. The whole big dynamical system for all magnitudes $X_i$ consists then of $M_a$ almost independent (up to exponentially small corrections) ${\bar{n}}$-dimensional dynamical systems. This big system has then at least $2^{M_a}$ different local attractors.

#### Transformation of chaotic time dynamics into developmental programs

In this subsection, we describe how chaotic (or stochastic) gene signals in time can be transformed into cellular patterns of terminal differentiation in space. Let us state first a formal mathematical definition of the cell developmental program. Such programs transform smooth morphogenetic fields into discrete cell patterns.

Let us consider strings $s=(s_1, s_2, ..., s_{m})$ consisting, for example, of symbols $s_j \in \{r, b\}$ (an extension to larger alphabets is straightforward). The set of strings ${{\mathscr {S}}}_D=\{ s^{(1)}, s^{(2)}, ..., s^{(m)} \}$, the time moments $t_j=j\Delta T$ and points ${\bar{x}}_1< {\bar{x}}_2< \cdots < {\bar{x}}_n$, where $j=1,..., n$, can be called a developmental program if the morphogen values take prescribed values at given time moments $t_k$ at given points ${\bar{x}}_j$:

6 $$\begin{aligned} {{\mathscr {M}}}(u(\cdot , t_k)) ({\bar{x}}_j) = m({\bar{x}}_j, t_k)=s^{(k)}_j, \quad \forall k,j, \end{aligned}$$

where a morphogenetic operator ${{\mathscr {M}}}$ transforms 1*D* patterns *u*(*x*) into strings *s* of the length *n*. This formal definition is illustrated by Fig. 1. In the simplest case, similarly to the French flag model, one has ${{\mathscr {M}}}(u(\cdot )) ({\bar{x}}_j)=r$ if $u({\bar{x}}_j)>u_0$ and ${{\mathscr {M}}}(u(\cdot )) (\bar{x}_j)=b$ otherwise. For a more complicated example connected with a partition of the phase space of morphogen concentrations, see Fig. 2.

We use the results of the work^11–13^ and it can be described as follows. The idea can be illustrated by Fig. 1. The left column of the cells is a string $\textbf{a}_1=a(1, t_1) a(1, t_2) \cdots a(1, t_K)$, $a_i \in \{r, b\}$, the next one is a string $\textbf{a}_2$, and the last column is a string $\textbf{a}_{N_c}(K)$, where $N_c$ is the number of the cells, *K* is the number of steps in time, and $a(j, t_k)$ denotes the type of the cell emerged at *j* position along *x*-axis at the moment $t_k$. Each column (the string, or the 1*D*-pattern) can be generated by a TM. In turn, these TMs can be simulated by few-dimensional chaotic dynamical systems^11,12^. This dynamical system is defined by interactions between the amplitudes $X_{i}(t)$ corresponding to the *k*-th segment group (see the previous subsection). So, we set $M_a=N_c$, further we take $M_a$ chaotic dynamical systems and each system generates the prescribed string (the column on Fig. 1) at a moment in time.

Let us consider first how to generate 1*D*-patterns consisting of the cells of two types, say, red and blue cells. Imagine an arbitrary string of cells of different types located along the *x*-axis (this might model 1*D*-organisms, like a worm, or a segmented embryo, see Fig. 1). Let *w* be a morphogen and its concentration is bounded, $w \le w_{+}$. Suppose the red cells appear when the average concentration *w* in the cell lies in $W_r=(0, w_{+}/2)$ and the blue cells emerge when this concentration is more than $w_{+}/2$: $w \in W_b=(w_{+}/2, w_{+})$. Therefore, to generate such strings, we should satisfy conditions ${\bar{w}}(j, t_k) \in W_{a(j,t_k)}$ for all $j=1,..., N_c, \ k=1,..., K$, where ${\bar{w}}(j, k)$ is the morphogen concentration averaged over *j*-th cell at the moment $t_k$ of cell differentiation. Suppose that *u*-reagent serves as a source of the morphogen *w*: $d_w \Delta w - b^2 w=u-u_0$, where the parameter $\beta =b{d_w}^{-1/2}$ determines the rate of decreasing *w*-concentration in space and $u_0(x,y)$ is a function. We adjust this constant in an appropriate way. The conditions ${\bar{w}}(j, t_k) \in W_{a(j,t_k)}$ can be satisfied if the time evolution of the amplitudes $X_j(t)$ corresponding to segments located at the *j*-th cell is governed by chaotic (or stochastic) dynamics. Note that the precise choice of this dynamics plays no role, but the corresponding attractor should be ergodic and mixing^13^.

## Discussion

The phenotype morphogenesis and its robustness are considered. The pattern formation robustness problem initiated by seminal papers^15,16^ was under intensive discussion last decades^17,18,20,42,43^ among many others, see the review^21^. A number of different approaches have been proposed: heat shock proteins and other molecular chaperones, methylation, microRNAs, emergent or embedded mechanisms, which are based on gene regulation network properties^18,42,44^ and nonlinearities in development.

The approach, proposed in this paper, looks directly opposite to many previous ones, where the formation of targeted patterns and robustness are achieved by a delicate tuning of gene network properties^42,44,45^. Based on Turing machine theory and the results of dynamical system theory, we come to counter-intuitive conclusions: a delicate tuning of reaction parts or network interconnections is not obligatory to generate complex target patterns and attractors in a robust manner. The general idea can be formulated as follows: *timing instead of tuning*. We can take any dynamical system, which generates complicated Turing complete dynamics (now we know a number of such systems) and then, if the target pattern is predetermined, this dynamics generates this pattern. The mechanism of pattern formation is robust with respect to noise and other perturbations if it goes in several stages, each of which, upon reaching a certain terminal state, ends up (similarly to Turing machines) with a stop signal. Such multistage morphogenesis, where each stage finishes with a stop signal, is robust with respect to perturbations. The idea that evolution can use any means to produce effective structures (evolutionary tinkering) was proposed by Jacob^6^. Here, we assert that practically any dynamical system with complicated large-time behavior can be used to generate complicated patterns.

For popular reaction-diffusion models, our mechanism of complex pattern and attractor generation is based on two fundamental ideas: a generalisation of Turing instability^2^, and on the Wolpert concept of positional information^1^. Note that the idea to use spatial heterogeneity was proposed still in the paper^2^. One can take sufficiently general chemical interaction and just adjust the correct positional information. Note that such robust morphogenesis can be performed via practically arbitrary chemical dynamics (if we adjust correctly spatially extended gradients). In fact, we show that if Wolpert’s and Turing’s pattern formation mechanisms work together then they can create, in a practically arbitrary nonlinear chemical reactor (corresponding to an open chemical system), complicated attractors of high complexity and even produce a number of such attractors and the corresponding cell patterns.

Note that the experimental data show that many genes exhibit an oscillatory behavior^46,47^. A model describing the chaotic behavior of transcription factors (TFs) is proposed in the research^47^, where it is shown that such a chaotic behavior could be useful for survival and leads to the formation of heterogeneous cell populations. Note that oscillatory gene dynamics was used to describe somitogenesis, the well-studied examples of pattern formation in the developing embryo are presented here^7,8,46^ and the first mathematical model is introduced here^48^. According to a popular formulation of this problem appearing in encyclopedias such as Wikipedia, “Somites are bilaterally paired blocks of paraxial mesoderm that form along the anterior–posterior axis of the developing embryo in segmented animals. In vertebrates, somites give rise to skeletal muscle, cartilage, tendons, endothelium, and dermis”. Although at an initial stage, somites manifest a periodical layered structure really the intrinsic states of cells are not periodic (otherwise, it would be impossible to obtain complex organs with further development). So, the true pattern is not periodic, and we believe, that instead of time-periodical dynamics chaotic dynamics should generate such non-periodicity in space-layered patterns.

These results show that there are chemical systems, which can be considered in the light of the analogy of Universal Turing machines (UTMs). A UTM can make all computations, which can be done by other TMs, and so, UTMs generate all possible string outputs when we vary their input. In our case, we have fixed (up to a few parameters to adjust) spatially extended systems, which, depending on initial data, generate different developmental patterns. So, open chemical systems of fairly general form serve as Universal Generators of spatiotemporal patterns and that generation is based on a complicated gene expression, chaotic or stochastic. A few genes are sufficient to encode those UPGs. Therefore, it is natural to expect that such chemical media could appear as a result of biological evolution.

## Supplementary Information

Supplementary Information.

## Data Availability

All data generated or analyzed during this study are included in this published article and its supplementary information files.
