# Supplementary material for: Robust morphogenesis by chaotic dynamics
Source: Sci Rep. 2023 May 9;13:7482. doi: 10.1038/s41598-023-34041-x (PMC10170119; doi:10.1038/s41598-023-34041-x)
Supplement: Supplementary file 1 — Supplementary Information. [file 41598_2023_34041_MOESM1_ESM.pdf]

Supplementary material to the manuscript "Robust morphogenesis by chaotic dynamics" (J. Reinitz, S. Vakulenko, I. Sudakow, and D. Grigoriev) published in Scientific Reports, 2023

Reaction-diffusion model defined by the system of equations (**1**) is considered by [1]. To simplify the statement we restrict ourselves to the case of the following two-component system:

$$u_t = d_u \Delta u + f(u, v) + \zeta, \tag{A1}$$

$$v_t = D_v \Delta v + h(u, v) + \eta, \tag{A2}$$

where $u = u(x, y, t)$ and $v(x, y, t)$ are unknown functions defined on $\Omega \times \{t \geq 0\}$, Ω is the strip $(-\infty, \infty) \times (0, 1) \subset \mathbf{R^2}$, D_v and d_u are diffusion coefficients for reagents u and v, respectively, $\eta(x, y)$ and $\zeta(x, y)$ are continuous functions, which can be interpreted as external sources independent of u, v. We assume

$$h(u, v), f(u, v) \in C^3. \tag{A3}$$

The initial conditions are given by

$$v(x, y, 0) = v^0(x, y), \quad u(x, y, 0) = u^0(x, y), \tag{A4}$$

where $u^0, v^0 \in C^0(\Omega)$. To simplify the problem, we set the periodic boundary conditions

$$v(x, y, t) = v(x + 2\pi, y, t), \quad u(x, y, t) = u(x + 2\pi, y, t), \tag{A5}$$

assuming that all the functions ζ, η, u^0 and v^0 are 2π -periodic in x. At the boundaries $y = 0$ and $y = 1$ we set the zero Dirichlet conditions for v, i.e.,

$$v(x, 1, t) = v(x, 0, t) = 0 \tag{A6}$$

and the zero Neumann conditions for u:

$$u_y(x, y, t)\Big|_{y=1, y=0} = 0. \tag{A7}$$

1 Dynamical complexity

Following [1] let us consider system (**2**), (**3**) with $d_u << D_v$. Our results hold under fairly general conditions to f, h, which we formulate below.

1.1 Assumptions to f and h

Suppose that

(**Bi**) *There exist u_* and v_* such that*

$$f_u(u_*, v_*) = 0, \quad f_v(u_*, v_*) = 0 \tag{A1}$$

and

$$h_u(u_*, v_*) \neq 0; \tag{A2}$$

(**Bii**) *The critical point u_*, v_* is non-degenerate, i.e., the Hessian H_f of the function f at the point (u_*, v_*) defined by*

$$\begin{pmatrix} f_{uu}(u_*, v_*) & f_{uv}(u_*, v_*) \\ f_{uv}(u_*, v_*) & f_{vv}(u_*, v_*) \end{pmatrix}$$

satisfies

$$\det H_f \neq 0. \tag{A3}$$

Let us consider certain examples. In many chemical and biological applications we are dealing with quadratic f and g, for example,

$$f(u, v) = c_{11}u + c_{12}v + \frac{f_{11}}{2}u^2 + f_{12}uv + \frac{f_{22}}{2}v^2, \tag{A4}$$

$$h(u, v) = c_{21}u + c_{22}v + \frac{h_{11}}{2}u^2 + h_{12}uv + \frac{h_{22}}{2}v^2. \tag{A5}$$

In this case assumptions **Bi** and **Bii** become

$$f_0 = det\ H = f_{22}f_{11} - f_{12}^2 \neq 0, \tag{A6}$$

$$u_* = f_0^{-1}(c_{12}f_{12} - c_{11}f_{22}) > 0, \tag{A7}$$

$$v_* = f_0^{-1}(c_{11}f_{12} - c_{12}f_{11}) > 0, \tag{A8}$$

and

$$c_{21} + h_{11}u_* + h_{12}v_* \neq 0. \tag{A9}$$

If $f_{11} = f_{22} = 0$ these conditions take the form

$$f_{12} \neq 0, \quad c_{12}f_{12} < 0, \quad c_{11}f_{12} < 0. \tag{A10}$$

Moreover, for chemical or biological applications it is natural to assume that reagent concentrations stay positive if they are positive at the initial moment. This means that the positive cone $\mathbf{R}^2_>$ is invariant under the corresponding semiflow. Then $c_{12} > 0$ and, therefore, conditions (A10) reduce to $c_{12} > 0$, $f_{12} < 0$ and $c_{11} > 0$.

In [1] the Brusselator is considered, which gives an example with cubic nonlinearities and which is an important theoretical model of an autocatalytic reaction. This system exhibits Turing instability and describes complicated spatial

patterns [2]. It can be shown that our assumptions **Bi** and **Bii** hold for this classical model [1].

Note that conditions **Bi** and **Bii** exclude monotone and gradient systems, which do not exhibit a stable chaotic large-time behavior. In fact, for gradient systems $D_v f = g_u$ thus conditions (A1) and (A2) cannot be fulfilled simultaneously.

To see a connection between assumptions **Bi**, **Bii** and conditions of monotonicity, let us consider the following class of systems with the parameters $\lambda, d, D, \eta(\cdot,\cdot)$ and $\zeta(\cdot,\cdot)$:

$$u_t = d_u \Delta u + f(u,v) - \lambda_1 u + \zeta(x,y), \tag{A11}$$

$$v_t = D_v \Delta u + h(u,v) - \lambda_2 v + \eta(x,y), \tag{A12}$$

under boundary and initial conditions (A4) -(A7). Let consider λ_i, d_u, D_v and functions η, ζ as parameters P of this family of the semiflows. Then the initial boundary value problems (IBVPs) are defined by eqs. (A11), (A12), boundary conditions (A5), (A6), (A7) and initial conditions (A4) generate a family of local semiflows $S^t(P)$, where each semiflow depends on the parameters P.

Since the coefficients λ_i are in the parameter list, we can remove the first condition (A1). Indeed, for any u_*, v_* we can set $\lambda_1 = f_u(u_*, v_*)$. Therefore, we conclude that the family of semiflows defined by IBVPs (A11), (A12), (A4) -(A7) has the property of universal dynamical approximation (see SM and [3]) under the following conditions: there exists a (u_*, v_*) such that

$$f_v(u_*, v_*) = 0, \quad g_u(u_*, v_*) \neq 0, \quad H_f(u_*, v_*) \neq 0 \tag{A13}$$

or there exists a (u_*, v_*) such that

$$h_u(u_*, v_*) = 0, \quad f_v(u_*, v_*) \neq 0, \quad H_g(u_*, v_*) \neq 0. \tag{A14}$$

Note that conditions (A13) and (A14) admit a transparent chemical interpretation: one of the reagents (u, v) is neither an activator nor an inhibitor for the other reagent.

1.2 Theorem on attractor complexity

Let us formulate the main result on the attractor complexity. To this end, let us remind Theorem on the Persistence of hyperbolic sets (for definitions of topological orbital equivalency, hyperbolic dynamics and structural stability see [4]).

Roughly speaking, hyperbolic dynamics (which may be periodic or even chaotic), is stable under small perturbations: if Γ is a hyperbolic set for a flow S^t on a compact smooth finite dimensional manifold, then for any C^1 -close flow $\tilde{S}^t$ there exists a hyperbolic set $\tilde{\Gamma}$ close to Γ and carrying the same dynamical behavior. The last fact means that there exists a homeomorphism h close to identity, which transforms trajectories of S^t on Γ onto trajectories of $\tilde{S}^t$ on $\tilde{\Gamma}$ (this homeomorphism performs orbital topological equivalency). Since h is close

to identity, the distance $dist(\Gamma, \tilde{\Gamma})$ between Γ and $\tilde{\Gamma}$ is small. Recall that for two subsets A, B of a Banach space $\mathbf{B}$ with a norm $|| \, ||$ the distance $dist(A, B)$ is defined by

$$dist(A, B) = \max\left\{ \sup_{x \in A} \inf_{y \in B} ||x - y||, \ \sup_{y \in B} \inf_{x \in A} ||x - y|| \right\}. \tag{A15}$$

Theorem I *Suppose assumptions* **Bi** *and* **Bii** *are satisfied. Then the family of the local semiflows* $S^t_{\mathbf{P}}$ *generates all finite-dimensional hyperbolic dynamics (up to orbital topological equivalencies) as we vary the parameters* $\mathbf{P}$ *of the IBVP defined by eqs. (2), (3) and conditions (A4) -(A7).*

The solutions u, v can be described explicitly. Namely, there are functions $u_0(x, y)$, $v_0(x, y)$ and $\theta_i(x, y)$, $V_i(x, y)$, $i = 1, \ldots, N$ such that

$$u = u_0 + \gamma \sum_{i=1}^{N} X_i(t)\theta_i + \tilde{u}, \tag{A16}$$

$$v = v_0 + \gamma \sum_{i=1}^{N} X_i(t)V_i + \tilde{v}, \tag{A17}$$

where $X_i(t)$ are functions of t, $\gamma > 0$ is a small parameter, and $\tilde{v}, u_1$ are small corrections such that $||\tilde{v}||_\alpha, ||\tilde{u}||_\alpha < c\gamma^{s_0}$, with $s_0 > 1$ and $\alpha \in (3/4, 1)$. Here $||f||_\alpha$ denotes the standard norm in fractional Banach spaces associated with our problem (for a definition of these spaces see, for example, [5]):

$$||f||_\alpha = ||(-\Delta)^\alpha f||_{L_2(\Omega)}.$$

The admissible choice of the numbers N is defined by on d_u and D_v: $1 \le N < N_0(d_u, D_v)$, where the integer N_0 goes to ∞ as $d_u \to 0$ under the condition $D_v > 1$.

Then, as a result of time rescaling $\tau = \gamma^s t$ with a $s > 0$ and removing small correction terms, we obtain the following system of differential equations with quadratic nonlinearities for $X_i(t)$:

$$\frac{dX_i}{d\tau} = \sum_{j=1}^{N} M_{ij} X_j + c X_i^2, \tag{A18}$$

where a constant c does not depend on small parameters, and a square $N \times N$ matrix $\mathbf{M}$ with entries $M_{ij} = M_{ij}(\eta, \zeta)$ depends, in a complicated way, on the functions η and ζ. Nonetheless, one can prove the following claim, which is a key point in the proof of Theorem I [1].

Control Lemma I. *For each* $\delta > 0$ *and each square* $N \times N$ *matrix* W_{ij} *there exist smooth* η *and* ζ *such that*

$$|W_{ij} - M_{ij}(\eta, \zeta)| < \delta \quad i, j \in [1, N], \tag{A19}$$

where $[1, N]$ denotes the set $\{1, 2, ..., N\}$. One can show [6] that when we change the number N and the matrix $\mathbf{M}$, the quadratic systems (A18) realize a dense set in the space of all finite-dimensional smooth vector fields defined on unit balls, i.e., the family of systems (A18) enjoys the property of universal dynamical approximation (UDA) (about UDA, see subsection 1.7, and [3]). This fact implies Theorem I, which asserts that the large time dynamics of $X_i(t)$ can be complicated when the problem parameters are adjusted in a special way.

Consider systems (A11), (A12) under boundary and initial conditions (A4) - (A7). For these IBVPs, we obtain, as a corollary of Theorem I, that either they generate monotone semiflows, or these systems can generate all structurally stable dynamics when we vary their parameters. In fact, if condition (A13) is not fulfilled, then f_v does not change its sign. On the other hand, if (A14) does not hold, g_u does not change its sign. Therefore, if conditions (A13) and (A14) are not satisfied, both f_v and g_u do not change their sign and therefore, IBVP is defined by eqs. (A11), (A12) under conditions (A4) -(A7) generates a monotone semiflow [7]. Finally, we obtain that either our model generates monotone semiflows only, or these systems can generate all structurally stable dynamics when we vary their parameters. Note that Theorem I can be extended on the case of dimension $d = Dim\Omega > 2$, however, it is not known whether Theorem I is true for $d = 1$.

1.3 Chemical-biological interpretation

Theorem I can be explained by non-formal arguments. The spatially heterogeneous sources $\eta(x, y)$ and $\zeta(x, y)$ create a non-trivial spatially heterogeneous non-perturbed pattern $(u_0(x, y), v_0(x, y))$, and for all $t > 0$ the solutions $(u(x, y, t), v(x, y, t))$ stay in a small neighborhood of that pattern. Inside this neighborhood, the dynamics $S^t(P)$ is weakly nonlinear: it is governed by an evolution equation, which involves a linear operator $\mathbf{A}$ and small nonlinear terms [1]. Adjusting appropriate η, ζ we obtain that the spectrum of $\mathbf{A}$ has a remarkable structure: it contains N of eigenfunctions $\psi_i = (\theta_i, V_i)^{tr}$ with small eigenvalues and all the remaining spectrum lies in a negative left half-plane and it is separated by a non-small barrier $b > 0$ from away the imaginary axis. On the physical language, we have N slow modes and an infinite number of fast modes. It is well known that the large-time dynamics of such slow-fast systems is captured by dynamics of slow modes [8]. The crucial fact is that we can control the number of slow modes N and a small interaction between those mode amplitudes $X_i(t)$ via the spatially heterogeneous sources $\eta(x, y)$ and $\zeta(x, y)$ (see Control Lemma I formulated above). This control property, which holds only for dimensions $d \geq 2$, allows us to prove that our semiflow $S^t(P)$ has the property of universal dynamical approximation.

This picture can be translated into biological language. Consider external sources $\eta(x, y)$ and $\zeta(x, y)$ in (**2**), (**3**) as spatial concentrations of certain morphogens. To obtain a complicated attractor, we need complicated functions $\eta(x, y)$ and $\zeta(x, y)$. We can thus consider those functions as carriers of the positional information that permits us to create complicated spatiotem-

poral patterns. Moreover, we note that in order to have a complex pattern $u(x, y, t), v(x, y, t)$ we need small coefficients d_u and $D_v >> d_u$, i.e., one reagent should diffuse much faster than the other one. Under this condition, the positional information can be transformed into a spatiotemporal structure with complex large-time behavior. So, our mechanism to generate complex patterns is based on the Wolpert concept of positional information [9] and the Turing approach [10]. Note that the idea to use spatial heterogeneity was proposed still in [10].

1.4 Generation of a number of local attractors

In this subsection, we state a new result with respect to [1]. Consider the following relation for M_{ij} obtained in [1]:

$$M_{ij} = \sum_{m=1}^{\infty} b_m \hat{g}_{mj} \exp\big(-m|x - \tilde{x}_i|\big), \tag{A20}$$

where $b_m = 1 - (-1)^m$, the coefficients $\hat{g}_{mj}$ depend on the problem parameters P and we can adjust the values of those coefficients in an arbitrary way as well as the points $\tilde{x}_1 < ... < \tilde{x}_N$ such that

$$\min_{i=1,\dots,N-1} |x_i - x_{i+1}| > \epsilon^{1/2}, \quad d_u = \epsilon^2, \tag{A21}$$

where $\epsilon > 0$ is a small parameter. We set $N = N_c\tilde{M}$, where $\tilde{M}$ and N_c are integers and decompose the points $\tilde{x}_j$ into N_c groups $G_1, ..., G_{N_c}$. The points of k-th group G_k we denote by $x_{i,k}$. The key idea in the choice of these groups and the points is that the points inside the same group are close while the groups are separated. Namely,

$$diam\ G_k = O(\epsilon^{1/2}), \quad dist\{G_k, G_l\} = O(1) \quad k \neq l, \tag{A22}$$

as $\epsilon \to 0$. Moreover, we set

$$m = m_0 \lfloor \epsilon^{-1/2} \rfloor, \quad m_0 \in \{1, 2, ..., \tilde{M}\}, \tag{A23}$$

where $\lfloor x \rfloor$ stands for the floor of x. Then for all $i, j \in [1, \tilde{M}]$ one has

$$\exp\big(-m|x_{i,k} - x_{j,k}|\big) = O(1), \tag{A24}$$

$$\exp\big(-m|x_{i,k} - x_{j,l}|\big) < \exp\big(-c_0\epsilon^{-1/2}\big), \quad (k \neq l) \tag{A25}$$

as $\epsilon \to 0$. Then we obtain that system (A18) can be decomposed into N_c independent (up to exponentially small smooth corrections) systems

$$\frac{dX_{i,k}}{d\tau} = \sum_{j=1}^{\tilde{M}} M_{ij}^{(k)} X_{j,k} + cX_{i,k}^2. \tag{A26}$$

The matrices $M_{ij}^{(k)}$ depends on the parameters P and for them an analog of control Lemma holds. Therefore each system (A26) realizes a dense subset of the set of all vector fields defined on the balls $\{||X^{(k)}|| < R\}$, where $X^{(k)}$ is the vector with components $X_{j,k}$, $j = 1, ..., \tilde{M}$ and R is an appropriate constant (on realizations of vector field see Appendix). Therefore, we can choose $\tilde{M}$ and $M_{ij}^{(k)}$ such that k-th system (A26) realizes a structurally stable smooth dynamical system

$$dq/dt = Q^{(k)}(q) \tag{A27}$$

on 3-dimensional ball $\mathbf{B}^3_{R_0}$ of the radius $R_0 > 0$ centered at $q = 0$ and having two local attractors. The first one is a rest point $\{0\}$ and the second one is a hyperbolic chaotic attractor $\mathcal{A}_k$. Note that each sequence $A_1A_2...A_{N_c}$, where A_k is either $\{0\}$, or $\mathcal{A}_k$ corresponds to a local attractor of the system (A18). This shows that the system (A18) has at least 2^{N_c} local attractors, which are Cartesian products of local attractors for subsystems. The number N_c admits estimate $N_c \geq M_a$. This observation implies the assertion (**i**) of Claim I for two-component systems when $m = 2$ and thus for all $m \geq 2$.

To show assertion **ii**, we can consider a system composed of $\tilde{m} = \lfloor m/2 \rfloor$ independent subsystems of the form (**1**). If each subsystem has M local attractors, then the subsystem has $M\tilde{m}$ local ones. Let us note that weak interactions between subsystems do not violate this property. This observation finishes the proof of **II**.

1.5 Morphogenesis algorithm

We assume that j-th cell occupies a subdomain $\Omega_j = \{[\bar{x}_i - \delta_c, \bar{x}_j + \delta_c] \times [0, 1]\} \subset \Omega_L$ centered at $\bar{x}_j$, where $\bar{x}_j = \delta_c/2 + (j-1)\delta_c$. We thus have $M = L/\delta_c$ equidistant cells, $j = 1, ..., N_c$. The output cell pattern can be considered as a string s_{out} in the binary alphabet $\mathcal{K}_c = \{0, 1\}$: $s_{out} = \{a_1a_2...a_{N_c}\}$, where N_c is the number of cells and a_i denotes the cell type for the cell located at $\bar{x}_i$.

Let us describe now how the cell pattern can be produced in our model. Let us consider organisms consisting of two cell types a_1 and a_2 (the case of a greater number of cell types can be studied in a similar way). We can encode these binary cell types setting $a_1 = 0$ and $a_2 = 1$. We follow classical ideas [10, 9]. The cell pattern is a result of terminal differentiation, which goes by morphogens. Suppose $w(x, y, t)$ is the concentration of a morphogen. Following the positional information concept, one can assume that the cell type depends on the morphogen level. We can suppose that the cell-centered at $\bar{x}_j$ accepts the state a_l at the moment t_k if $\int_0^1 w(\bar{x}_j, y, t_k)dy \in W_{a_l}$, where W_0, W_1 two disjoint open sets in $\mathbf{R}$, which are subsets of the set of possible values of w.

Let $t_1 < t_2, ... < t_K$ be cell differentiation moments. For simplicity and taking into account the periodicity of internal cell dynamics, we assume that $t_k = k\Delta t$, $k = 1, ..., K$. It is convenient to introduce the following auxiliary functions of t:

$$Z_j(t) = \int_0^1 w(\bar{x}_j, y, t)dy, \quad j = 1, ..., N_c. \tag{A28}$$

Then, to generate a prescribed cell developmental program encoded by the matrix $\mathbf{A}$ with binary entries $a(l,m) \in \{0,1\}$, we should satisfy the following conditions:

$$Z_j(t_m) \in W_{a(j,m)}, \quad j = 1, ..., N_c, \quad m = 1, ..., K, \tag{A29}$$

where $a(l,m) \in \{0,1\}$.

We proceed as follows. First, we set

$$w_t - \Delta w + \beta w = u - u_0, \tag{A30}$$

where $\beta > 0$ and w satisfies boundary conditions (A5), (A6). Taking into account asymptotics (A16) for u we obtain (up to small terms) that for large t

$$w(x,y,t) \approx C_{\gamma,\epsilon} \sum_{j=1}^{\tilde{M}} \sum_{k=1}^{N_c} X_{j,k}(t) \exp(-\beta|x - x_{j,k}|), \tag{A31}$$

where $C_{\gamma,\epsilon}$ is a constant. Using the last relation let us compute $Z_j(t)$ defined by (A28). We obtain then

$$Z_j(t) \approx C_{\gamma,\epsilon} \sum_{l=1}^{\tilde{M}} \sum_{k=1}^{N_c} X_{l,k}(t) \exp(-\beta|\bar{x}_j - x_{j,k}|). \tag{A32}$$

Suppose $\beta >> 1$ and $\beta << \epsilon^{-1/2}$. The points $x_{j,k}$, which lie in the same k-th group G_k, are $\epsilon^{1/2}$-close each to the other. On the other hand, the distances between different groups G_k have the order $O(1)$ and do not depend on ϵ. We choose $\bar{x}_j$ such that $\bar{x}_j$ is close enough to j-th group G_j and $dist(\bar{x}_j, G_k) = O(1)$ for $j \neq k$. Then for small $\epsilon > 0$ we can simplify (A32) that gives

$$Z_j(t_m) \approx C_{\gamma,\epsilon} \sum_{l=1}^{\tilde{M}} X_{l,j}(t_m). \tag{A33}$$

Let us fix j. The dynamics of $X_{l,j}(t)$ from j-th group is defined by j-th system (A27). This means that for large times t all the variables $X_{l,j}(t)$ depend on t via q thus $Z_j(t) \approx Z_j(q(t))$. Then (A29) can be rewritten as

$$q(t_m) \in V_{j,m}, \quad m = 1, ..., K, \tag{A34}$$

where

$$dq/dt = Q^{(j)}(q) \tag{A35}$$

and $V_{j,m}$ are some subsets of the ball $\mathbf{B}^3_{R_0}$ having non-zero Lebesgue measures. These sets are the inverse images (preimages) of the sets $W_{a(j,m)}$ under the maps $q \to Z_j(q)$.

In the next subsection, we will how to satisfy this relation (A34) and (A35) by chaotic dynamics.

1.6 Cell pattern algorithm

An algorithm to solve the pattern generation problems is based on celebrated results of dynamical system theory on hyperbolic sets, its persistence, the existence of Markov partitions and a connection between Bernoulli shifts and maps on invariant hyperbolic sets [11, 12, 13] can be found in [14].

Let us consider an important particular case of the Markov dynamics described above, when the Markov chain (process) is defined by the system of stochastic differential equations

$$dq = Q(q)dt + B(q)d\xi, \tag{A36}$$

where Q, B are smooth vector field defined on a ball $\mathbb{B}^n$ in $\mathbb{R}^n$, $\xi = (\xi_1, ..., \xi_p)$ are standard independent white noises. We suppose that the n-dimensional field Q is directed toward to the interior of $\mathbb{B}^n$ at its boundary, and B is a p-dimensional field vanishing at the boundary of that ball. This chain is a process with continuous time, but we can transform it into the discrete-time chain by the Poincaré sections transversal to the flow of the system (A36). Then we obtain a randomized Poincaré map.

We suppose that for $B = 0$ (in the absence of noise) eq. (A36) defines a smooth dynamical system defined with an attractor Γ, which has an invariant measure μ defined on Γ (for axiom A attractors and Anosov diffeomorphisms such measures exist and they are well studied, they are called Sinai–Ruelle–Bowen (SRB) measures [15, 16]). Suppose that the flow S^t generated by system (A36) has a strong mixing property, i.e.

$$\mu(S^t A \cap B) \to \mu(A)\mu(B) \quad t \to +\infty$$

for two measurable sets A, B. Let $\mu(A_0)$ be a subset of non-zero μ-measure on Γ and V_0 be a small open neighborhood of A_0 in $\mathbb{B}^n$. Let $E_1, E_2, ..., E_{n_c}$ be a fixed partition of Γ and p be a fixed positive integer. Then the mixing property implies that if ΔT is large enough all the following intersections are non-empty:

$$B_{kj} = S^{j\Delta T} A_0 \cap E_k \neq \emptyset, \quad j = 1, ..., p, \; k = 1, ..., n_c,$$

In fact, according to the strong mixing property $\mu(B_{kj}) > 0$. Moreover, it is easy to see that

$$V_{kj} = S^{j\Delta T} V_0 \cap E_k \neq \emptyset, \quad j = 1, ..., p, \; k = 1, ..., n_c.$$

This shows that for any finite sequence $\{a_j\}$, $j = 1, ..., p$ of $a_j \in \mathcal{K}_c$ there is an open (possibly having a small measure) set of initial points $X(0)$ such that

$$S^{j\Delta T}(X(0)) \in E_{a_j}, \quad j = 1, ..., p.$$

Let us note that the sophisticated algorithm using Poincaré maps proposed in [14] has an advantage with respect to the simplified one described here: by the Bernoulli shifts and the Markov partitions, we can find the set of initial data

$q(0)$ in an explicit way. For our simplified algorithm it can be done by numerical simulations. We have checked it for the Lorenz system for q.

As an example of the simplified algorithm application, let us consider first how to create the French flag pattern (the standard illustration of Wolpert gradient approach).

Let all layers of the cell pattern have the same width as the string corresponding to the French flag, which is $s = (1, 2, 3)$. Let us take the noisy perturbed Lorenz system for $q = (q_1, q_2, q_3)$, which has the form

$$dq_1 = \sigma(q_2 - q_1)dt + \epsilon d\eta_1(t),$$
$$dq_2 = (q_1(\rho - q_3) - q_2))dt + \epsilon d\eta_2(t),$$
$$dq_3 = (q_1 q_2 - bq_3)dt + \epsilon d\eta_3(t),$$

where $\eta_i(t)$ are independent standard Wiener processes. We suppose that $\epsilon > 0$ is a small parameter, which defines the noise level. We make the standard choice of the parameters σ, ρ, b leading to a chaotic Lorenz attractor Γ ($\sigma = 10, \beta = 8/3$ and $\rho = 28$).

Let us introduce $q_{min} = \min_{q \in \Gamma} q_1$, $q_{max} = \max_{q \in \Gamma}$, and $\Delta q = q_{max} - q_{min}$. Then we make the partition $R_1 = \{q : q_1 \in (q_{min}, (q_{max} - q_{min})/2)\}$, $R_2 = \{q : q_1 \in (q_{min} + (q_{max} - q_{min})/2, q_{max})\}$. The cell fate is determined by the averaged signal

$$\bar{q}_1(t) = 2\tau^{-1} \int_{t-\tau}^{t+\tau} q_1(s)ds. \tag{A37}$$

at the moments $\Delta T, 2\Delta T...$ when the wave arrives at the cell. This time-averaging helps to repress fluctuation effects.

Our numerical algorithm is as follows. We use the standard Euler method with a small step, where at each step we generate random numbers $d\eta_j$. Numerical simulations show that there are starting points $q(0)$ and ΔT such that for example $\bar{q}_1(\Delta T) \in R_1$, $q(2\Delta T) \in R_2$, and $\bar{q}_1(3\Delta T) \in R_1$. So, we obtain the layered $1D$-pattern consisting of 3 layers encoded by the string 121. The same construction allows us to obtain more complicated patterns, for example, consisting of 5 and more layers. Let us note that one can take other sets R_k, however, these sets should intersect the Lorenz attractor so that the RBS measures of intersections are not too small. Note that the longer the cell pattern s becomes, the smaller the set A_0 of appropriate starting points $q(0)$ will be.

1.7 Realisation of vector fields (RVF)

The main technical tool in proving attractor complexity for partial differential equations and systems is the realization vector field (RVF) method using, in particular, structural stability ideas. It is based on a classical center manifold technique and on the well known idea that any n-dimensional dynamics can bifurcate from an equilibrium with n- zero eigenvalues if the number of bifurcation parameters is large enough. Such approach was used for finite dimensional systems (see [17]), but it can be extended on infinite dimensional evolution

equations. This RVF approach is developed by first P. Poláčik to prove the existence of non-trivial large-time behavior for quasilinear parabolic equations (see [18, 19]) and developed in [20, 1] for reaction-diffusion systems, in [21] for neural networks and in [22] for weakly compressible Navier-Stokes equations.

In our model, $n = N$, where N is the number of kinks, eigenvalues are exponentially close to zero and the initial data z_0 plays the role of the main bifurcation parameter, i.e., the bifurcation parameter is infinite-dimensional.

1.7.1 Structural stability

Recall the basic concept of structural stability introduced by A. Andronov and S. Pontryagin in 1937. Consider a smooth vector field Q on a compact domain $\mathbb{D}^n$ of $\mathbb{R}^n$ with a smooth boundary (or on a compact smooth manifold M of dimension n). Assume that $Q \in C^1(\mathbb{D}^n)$ and consider all δ-small perturbations $\tilde{Q}$ such that

$$|\tilde{Q}|_{C^1(\mathbb{D}^n)} < \delta. \tag{A38}$$

Consider systems of differential equations $dq/dt = Q(q)$ and $dq/dt = Q(q) + \tilde{Q}(q)$ and suppose that they define global semiflows S^t_Q and $S^t_{Q+\tilde{Q}}$ on $\mathbb{D}^n$. The system $dq/dt = Q(q)$ is called structurally stable if there exists a δ_0 such that if

$$|\tilde{Q}|_{C^1(\mathbb{D}^n)} < \delta_0,$$

then trajectories of semiflows S^t_Q and $S^t_{Q+\tilde{Q}}$ are orbitally topologically conjugate (there exists a homeomorphism, which maps trajectories of the first system into trajectories of the second one). Roughly speaking, the original system is structurally stable if any sufficiently small C^1 perturbations of that system conserve the topological structure of its trajectories, for example, the equilibrium point stays an equilibrium (maybe, slightly shifted with respect to the equilibrium of a non-perturbed system), the perturbed cycle is again a cycle (maybe, slightly deformed and shifted).

Note that structurally stable dynamics may be, in a sense, "chaotic". There is a rather wide variation in different definitions of "chaos". We restrict ourselves to hyperbolic chaotic sets. Chaotic (no periodic and no rest point) hyperbolic sets occur in some model systems [23, 4, 11].

1.8 RVF for evolution problems in Banach spaces

Let us consider a family of local semiflows $S^t_{\mathbf{P}}$ in a fixed Banach space B. Assume these semiflows depend on a parameter $\mathbf{P} \in B_1$, where B_1 is another Banach space. Denote by $\mathcal{B}^n(R)$ the ball $\{q : |q| \le R\}$ in $\mathbb{R}^n$, where $q = (q_1, q_2, ..., q_n)$ and $|q|^2 = q_1^2 + ... + q_n^2$. For $R = 1$ we will omit the radius R, $\mathcal{B}^n = \mathcal{B}^n(1)$. Remind that a set M is said to be locally invariant in an open set $W \subset B$ under a semiflow S^t in B if M is a subset of W and each trajectories of S^t leaving M simultaneously leaves W. In this paper, all W are tubular neighborhoods

of the balls $\mathcal{B}^n(R)$, which have small widths. Consider a system of differential equations defined on the ball $\mathcal{B}^n$:

$$\frac{dq}{dt} = Q(q), \tag{A39}$$

where

$$Q \in C^1(\mathcal{B}^n), \quad \sup_{q \in \mathcal{B}^n} |\nabla Q(q)| < 1. \tag{A40}$$

Assume the vector field Q is directed strictly inward at the boundary $\partial\mathcal{B}^n = \{q : |q| = 1\}$:

$$Q(q) \cdot q < 0, \quad q \in \partial\mathcal{B}^n. \tag{A41}$$

Then system (A39) defines a global semiflow on $\mathcal{B}^n$. Let δ be a positive number.

Definition (**realization of vector fields**) *We say that the family of local semiflows $S^t_{\mathbf{P}}$ realizes the vector field Q (dynamics (A39)) with accuracy $\delta > 0$ (briefly, δ - realizes), if there exists a parameter $\mathbf{P} = \mathbf{P}(Q, \delta, n) \in B_1$ such that*

(**i**) *semiflow $S^t_{\mathbf{P}}$ has a locally invariant in a open domain $\mathcal{W} \subset B$ and locally attracting manifold $\mathcal{M}_n \subset B$ diffeomorphic to the unit ball $\mathcal{B}^n$;*

(**ii**) *this manifold is embedded into B by a map*

$$z = Z(q), \quad q \in \mathcal{B}^n, \quad z \in B, \quad Z \in C^{1+r}(\mathcal{B}^n), \tag{A42}$$

where $r > 0$;

(**iii**) *the restriction of the semiflow $S^t_{\mathbf{P}}$ to $\mathcal{M}_n$ is defined by the system of differential equations*

$$\frac{dq}{dt} = Q(q) + \tilde{Q}(q, \mathbf{P}), \quad Q \in C^1(\mathcal{B}^n), \tag{A43}$$

where

$$|\tilde{Q}(\cdot, \mathbf{P})|_{C^1(\mathcal{B}^n)} < \delta. \tag{A44}$$

Definition 2. *Let Φ be a set of vector fields Q, where each Q is defined on a ball $\mathcal{B}^n$, positive integers n may be different. We say that the family $\mathcal{F}$ of local semiflows $S^t_{\mathbf{P}}$ realizes the family Φ if for each $\delta > 0$ and each $Q \in \Phi$ the field Q can be δ -realized by the family $\mathcal{F}$.*

We say that the family of global semiflows $\mathcal{F}$ has the property of universal dynamical approximation if that family realizes the set of all C^1- smooth finite dimensional fields defined on all unit balls $\mathcal{B}^n$.

Many systems enjoy the property of universal dynamical approximation, for example, the Lotka-Volterra system with many species, the Hopfield system, a large class of reaction-diffusion systems, and others. In the coming subsection, we will consider an example of applying the RVF method.

1.9 Realization of Rössler system by two component reaction diffusion systems

The well-known Rössler system is one of the simplest systems of ODEs exhibiting chaotic large-time behavior. It reads:

$$\frac{dX_1}{dt} = -X_2 - X_3, \tag{A45}$$

$$\frac{dX_2}{dt} = X_1 + aX_2, \tag{A46}$$

$$\frac{dX_3}{dt} = b + X_3(X_1 - c), \tag{A47}$$

where a, b, c are parameters. Under a choice of a, b, c, for example, $a = 0.2, b = 0.2$ and $c = 5.7$, the solutions of the Cauchy problem for this system are chaotic (it can be checked numerically).

Of course, there exist a number of other systems of ODEs generating chaos, for example, the famous Lorenz system. However, in opposite to the Lorenz system, the Rössler one appears in biochemistry. We will see that there exists an algorithm permitting to realize a system of eqs. (A45), (A46), and (A47) via a class of two-component reaction-diffusion systems. A similar algorithm also realizes all quadratic systems of ODEs (for example, the multispecies Lotka-Volterra systems) by these two-component reaction-diffusion systems.

Consider the following equations for two reagents u, v:

$$u_t = \epsilon^2 \Delta u + W(x)u + g_1(x)v + g_2(x)uv + h(x), \tag{A48}$$

$$v_t = D\Delta u - a_0^2 v + g(x, y)u, \tag{A49}$$

where the smooth functions W, g, g_k, h and ϵ, a_0, D are parameters $\mathbf{P}$. We consider equations (A48) and (A49) in the strip $\Pi = (-\infty, \infty) \times [0, L_2]$ under the following boundary conditions

$$u(x + L_1, y, t) = u(x, y, t), \quad v(x + L_1, y, t) = v(x, y, t) \tag{A50}$$

$$u_y(x, y, t)\Big|_{y=0,L_2} = 0, \quad v(x, y, t)\Big|_{y=0,L_2} = 0. \tag{A51}$$

Such a choice of boundary conditions simplifies the problem analysis permitting for quasi-one-dimensional layered solutions for u.

Assertion. *Under an appropriate choice of W, g_k and ϵ, a_0, D and initial conditions $u_0(x) = u(x, y, 0), v_0(x, y) = v_0(x, y, 0)$ the IBVP defined by eqs. (A48), (A49) and boundary conditions (A50), (A51) reduces to system (A45), (A46), and (A47).*

Below we outline the proof of this assertion illustrated by some numerical simulations for a finite difference approximation of the system (A48), (A49). The proof follows the ideas stated above and in [8, 24, 25, 1].

1.9.1 Construction of W, g and asymptotic solutions

The first key step is a choice of $W(x)$, where we use ideas well known in the theory of the Schrödinger operators (see, for example, [25]). Let us consider the 1D Schrödinger operator $\mathcal{H} = \epsilon^2 \frac{d^2}{dx^2} + W(x)$ on $(-\infty, \infty)$. We can adjust such potential W that this operator will have three eigenvalues λ_k and three well-localized eigenfunctions $\psi_k = \psi(\epsilon^{-1}(x - \bar{x}_k))$ with $0 < \bar{x}_1 < \bar{x}_2 < \bar{x}_3 < L_1$ corresponding to a discrete spectrum such that $\lambda_k = 0$, $k = 1, 2, 3$ whereas all the remaining spectrum is separated by a barrier $\sigma > 0$ from the imaginary axis and lies in the $(-\infty, -1)$, where $\sigma > 0$ is independent of ϵ. Note that since we consider the Neumann conditions at $y = 0, L_2$, the same eigenfunctions are "almost" eigenfunctions of the two-dimensional operator $\epsilon^2 \Delta + W(x)$, which satisfies periodic boundary conditions with an exponentially small accuracy $O\big(\exp(-c\epsilon^{-1})\big)$. It permits us to use them to construct an asymptotic solution (for technical details, see [1]).

The solution u can be then represented as

$$u(x, y, t) = X_1(t)\psi_1(x) + X_2(t)\psi_2(x) + X_3(t)\psi_3(x) + \tilde{u}(x, y, t), \tag{A52}$$

where X_i are slowly evolving amplitudes and $\tilde{u}$ is a small correction orthogonal to ψ_k in $L_2(\Pi)$. The standard ideas for slow-fast decomposition show then [8, 24, 1] that the v-component has the form

$$v(x, y, t) = V(x, y, X) + \tilde{v}, \tag{A53}$$

where $\tilde{v}$ is a small correction, and the main contribution V can be represented as sum of three terms:

$$V = V_1(x, y)X_1 + V_2(x, y)X_2 + V_3(x, y)X_3,$$

where

$$D\Delta V_k - a_0^2 V_k = g(x, y)\psi_k(x). \tag{A54}$$

Let us assume that $||\psi_k|| = 1$ and they are orthonormal. The time evolution of X_k is governed by a shorted Galerkin system:

$$\frac{dX_k}{dt} = \lambda_k X_k + \big(g_1 v + g_2 uv + h), \psi_k\big) \tag{A55}$$

where (f, g) denotes the standard inner scalar product in $L_2(\Pi)$. We can simplify (A55) by substituting asymptotics (A52) and (A53) into the right-hand side of (A55). Taking into account that ψ_k are localized in ϵ- neighborhoods at $\bar{x}_k$ we obtain (using the Fourier decomposition) the following asymptotics for V_k:

$$V_k(x, y) \approx \sum_{m=1} \hat{g}_{mk} \frac{\exp(-\bar{k}(m)|x - \bar{x}_j|)}{\bar{k}(m)} b_m \sin(m\pi y/L_2), \tag{A56}$$

where $\hat{g}_{mk}$ are the Fourier coefficients of $g(\bar{x}_k, y)$:

$$\hat{g}_{mk} = \int_0^{L_2} \sin(m\pi y/L_2) dy$$

and

$$k(m) = \sqrt{(m\pi/L_2)^2 + a_0^2}.$$

Then we obtain that (A55) reduces to the following quadratic system:

$$\frac{dX_k}{dt} = h_k + \lambda_k X_k + \sum_{l=1}^{3} A_{kl} X_l + X_k \sum_{l=1}^{3} B_{kl} X_l + r_k(X, \epsilon), \tag{A57}$$

where $k = 1, 2, 3$, functions $r_k(X, \epsilon)$ are small corrections and

$$A_{kl} = c_0 \epsilon^{1/2} \int_0^{L_2} g_1(\bar{x}_k) V_l(\bar{x}_k, y) dy, \quad h_k = c_1 \epsilon^{1/2} h(\bar{x}_k), \tag{A58}$$

$$B_{kl} = c_0 \epsilon^{1/2} \int_0^{L_2} g_2(\bar{x}_k) V_l(\bar{x}_k, y) dy, \tag{A59}$$

where c_0, c_1 are positive constants depending on the function $\psi(z)$. We obtain the Rössler system if

$$A_{11} = -\lambda_1,\ A_{12} = A_{13} = -1,\ A_{22} = -\lambda_2 + a,\ A_{21} = 1,\ A_{33} = -\lambda_3,\ A_{32} = A_{31} = 0, \tag{A60}$$

$$B_{ij} = 0,\ \forall i = 1, 2, j = 1, 2\ B_{31} = 1, B_{32} = B_{33} = 0,\ h_1 = h_2 = 0, h_3 = b. \tag{A61}$$

Therefore, the problem of realization reduces to the following: to adjust the Fourier coefficients $\hat{g}_k$ of g, and the functions h, g_1, g_2 so that the relations (A60) and (A61) hold. Due to the particular structure of these relations, this problem can be solved in a simple way. Let the support of h, g_2 be disjoint with intervals $(-\delta + \bar{x}_1, \delta + \bar{x}_1)$ and $(-\delta + \bar{x}_2, \delta + \bar{x}_2)$ and the support of a smooth g_1 is disjoint with $(-\delta + \bar{x}_3, \delta + \bar{x}_3)$. This choice provides that $h_1 = 0, h_2 = 0$ and $B_{ij} = 0$ for all $j = 1, 2, i = 1, 2$. Then relations (A60) and (A61) reduce to the following independent linear algebraic systems with respect to unknown Fourier coefficients:

$$\sum_{m}^{\infty} \frac{\exp(-\bar{k}(m)|\bar{x}_i - \bar{x}_j|}{\bar{k}(m)} b_m \hat{g}_{mj} = C_{ij}, \tag{A62}$$

where C_{ij}, $i = 1, 2, 3, j = 1, 2, 3$ are given numbers and $b_m = ((-1)^m - 1)/(m\pi/L_2)$ are integrals of $\sin(my\pi/L_2)$ over $[0, L_2]$, which do not equal zero for odd integers m.

For each j the corresponding system consists of three equations with a countable set of unknowns $\hat{g}_{mj}$. It is natural to seek solutions with the minimal norm $\sum_m |\hat{g}_{mj}|^2$. The numerical solution (see the next subsection) shows that such solutions can be obtained as follows. For each j we choose three odd integers m, say, $m_{1j} = 1, m_{2j} = 3, m_{3j} = 5$ or $m_{1j} = 1, m_{2j} = 3, m_{3j} = 7$. We set $\hat{g}_{m,j} = 0$ for all $m \neq m_{lj}$ and we are seeking for the coefficients $y_{l,j} = \hat{g}_{m_l j}$ by solving the linear algebraic systems with respect to $y_{l,j} = \hat{g}_{m_{lj} j}, l = 1, 2, 3$.

Comments.

(a) For the Rössler system, the construction is simpler than in the general case, although based on the same ideas. For example, the Lorenz system is much more difficult to realize via reaction-diffusion systems. It is connected with the fact that the Lorenz system has no biochemical interpretation, in opposition to the Rössler system.

(b) The chosen boundary conditions simplify the mathematical analysis because the Neumann conditions for u allow us to construct layered quasi-one-dimensional solutions for u. On the other hand, for v it is simpler to use the zero Dirichlet conditions at $y = 0, L_2$. In fact, for the zero Neumann conditions the corresponding eigenfunctions have the form $\cos(m\pi y/L_2)$ and the averages of these functions along $[0, L_2]$ equal zero. Then we are not capable to obtain arbitrary matrices A, B.

(c) The difference between this model and the classical approach starting with seminal Turing paper [10] is as follows. The classical morphogenesis theory for two-component models is based on the concept of the Turing pair, one reagent is an activator and the other is an inhibitor.

In this model, which involves spatial inhomogeneities, one of the reactants is neither an activator nor an inhibitor (it is a necessary condition to obtain a stable chaotic behavior in two-component systems, see [1] for mathematical details). To understand this situation, let us consider, for example, eq. (A49) and the reaction term $g(x, y)u$, which defines the production of v. The function g has a complicated form and changes the sign. In space domains, where $g > 0$, the reagent u serves as an activator, in domains, where $g < 0$, u works as an inhibitor for v. Similarly, for the equation (A48) with the term $(W(x) + g_2 v)u$. Here the reagent u is an activator in layers where $W(x) > -g_2 v$ and it is an inhibitor in other layers. So, we have a mosaic pattern of inhibition and activation. Such structure is necessary to provide a complex non-local interaction between X_1, X_2 and X_3, which can produce chaos (see the next comment).

(d) Physical principles are beyond mathematics.

The physical and biological ideas are quite standard and follow [26], where is shown a key role of local self-enhancement and long-range inhibition. In our model, local self-enhancement generates narrow vertical layers, where u is concentrated. In our model, we have three layers. The average u concentrations in layers are proportional to $X_1(t)$, $X_2(t)$ and $X_3(t)$. The layers are very narrow thus we have no essential direct interaction between layers. However, we have a long-range reagent v. The u acts to v creating the smooth pattern $V(x, y, X_1, X_2, X_3)$ (see Figs A1, A2). The new idea with respect to [26] is only the following. The mosaic structure of inhibition and activation induced by spatial gradients (described above) allows us to create complicated dynamics of X_i.

(e) Why there is an exponential number of local attractors? It is simpler to explain by the following simplified model, which is more tractable for numerical simulations and considered in the next subsection.

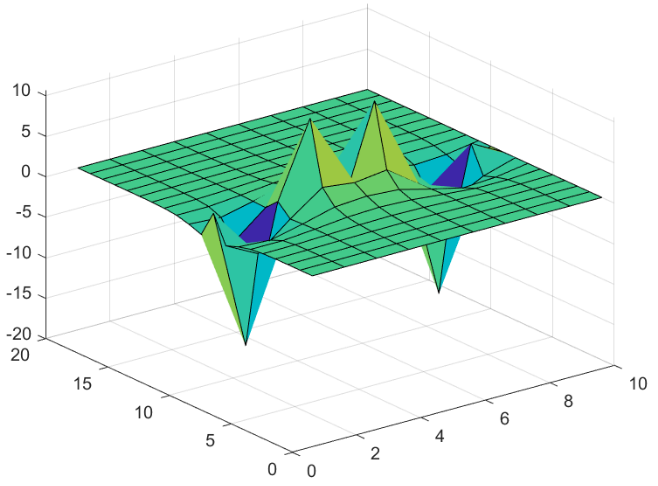

Figure A1: The plot shows the values of the function $V_1(x,y)$ on the $[0,L_1]\times[0,L_2]$ computed on the grid with the space step 1, i.e. for all positive integers (x,y).

1.9.2 Numerical simulations and images

Solutions of systems (A62) have the following form. For $j=1$ we take $m_1 = 1, m_2 = 3, m_3 = 7$ then

$$\hat{g}_{11} = 0.32, \quad \hat{g}_{31} = -6.98, \quad \hat{g}_{71} = 20.94,$$

for $j=1,2$ one has $m_1 = 1, m_2 = 3, m_3 = 5$,

$$\hat{g}_{12} = -0.268, \quad \hat{g}_{32} = 8.91, \; \hat{g}_{52} = -20.21,$$

and

$$\hat{g}_{13} = -0.478, \quad \hat{g}_{33} = 15.51, \quad \hat{g}_{53} = -32.34.$$

The functions $V_1(x,y)$, $V_2(x,y)$ and $V_3(x,y)$ are computed by these relations. V_1 and V_2 are shown in Fig. A1, A2.

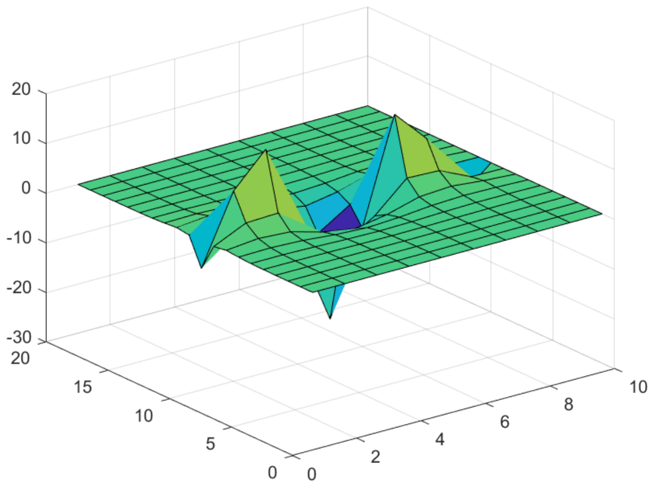

Figure A2: The plot shows the values of the function $V_1(x, y)$ on the $[0, L_1] \times [0, L_2]$ computed on the grid with the space step 1.

Making numerical calculations for singularly perturbed systems like (A48), (A49) is not an easy problem [25]. However, we can simplify the problem by replacing the equation (A48) with its simplest approximation on a two-dimensional lattice. For such an approximation, instead of sophisticated arguments from the Schrödinger operator theory, we can use a much more simple construction. The equation (A49) for v can be replaced by an integral representation by the Green function. Then we obtain a system of ordinary differential equations on the rectangular grid:

$$\frac{du(i,j,t)}{dt} = f(i)u(i,j,t) + V(i,j,t) + u(i,j,t)V(i,j,t) + h(i) + d(\Delta_2 u)(i,j,t), \tag{A63}$$

where $i \in \{1, 2, ..., L_1\}$, $j = \{1, 2, ..., L_2\}$, and

$$(\Delta_2 u)(i,j,t) = u(i,j-1,t) + u(i,j+1,t) - 2u(i,j,t),$$

with $u(i, 0, t) = u(i, L_2, t),\ u(i, L_2 + 1, t) = u(i, 1, t)$. From a biological point of view, we can consider these equations as a description of colonies of cells located at fixed positions interacting via a reagent v, which can diffuse between cells. Each cell produces a reagent u, which does not diffuse between layers of cells in x-direction.

For V we use the equation

$$V(x, y, t) = \sum_{i,j} G(x, y|i, j)g(i, j)u(i, j, t), \tag{A64}$$

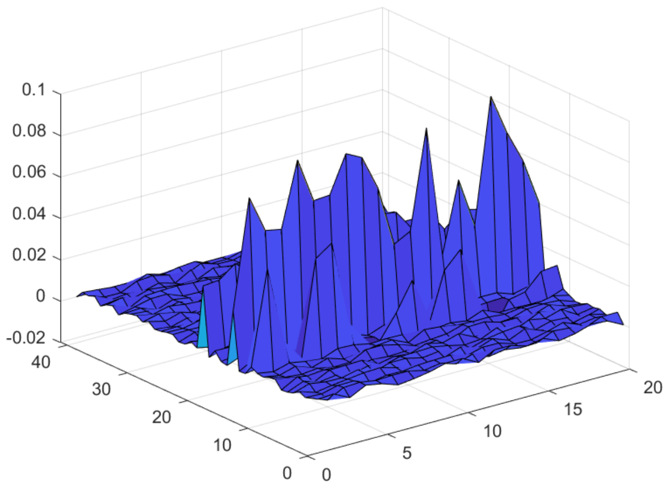

Figure A3: The plot shows the values of the function $u(i, j, t)$ on the grid $[0, L_1] \times [0, L_2]$ with the space step 1 for small $t = 0.4$. The grid size $L_1 = 40$, $L_2 = 20$. Solution of a system (A63), (A64) is found by the standard program ode45 in Matlab. The parameters are as follows. Let $i_1 = 12, i_2 = 15, i_3 = 20$ be positions for layer localization. $h_i = 0.2\delta(i - i_3)$, $i = 1, ..., L_1$, where δ is the discrete δ-function, $\delta(j) = 1$ for $j = 0$ and 0 otherwise, $f_i = -10$ for $i \neq i_k$, $f(i_1) = -1$, $f(i_2) = -1$, $f(i_3) = -5.7$, $g_1(i) = g_2(i) = 0$ for $i \neq i_k$, $g_1(i_1) = 1, g_1(i_2) = 1, g_1(i_3) = 0$ and $g_2(i_3) = 1, g_2(i_1) = 0, g_1(i_2) = 0$, $g(i, j)$ is constructed in a special way to simulate the Rössler system. The coefficient $D_2 = 0$.

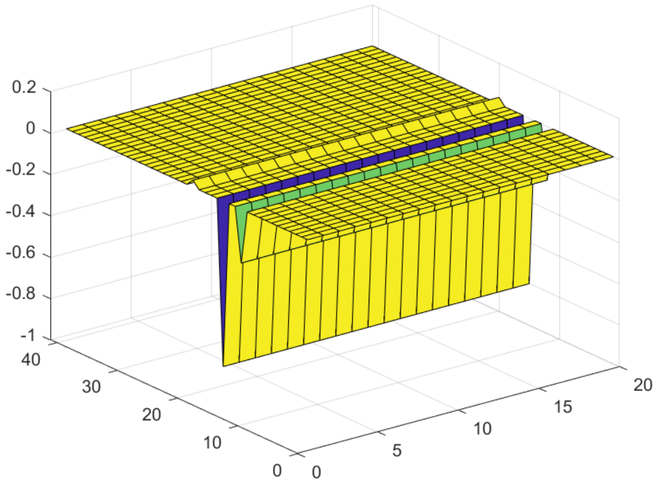

Figure A4: The plot shows the values of the function $u(i, j, t)$ on the grid $[0, L_1] \times [0, L_2]$ with the space step 1 for $t = 50$. Comparing this plot with the previous one we see that the solution u is localized in vertical layers $i = 12, 15, 20$ and it is almost one-dimensional, i.e. depends on i, t only.

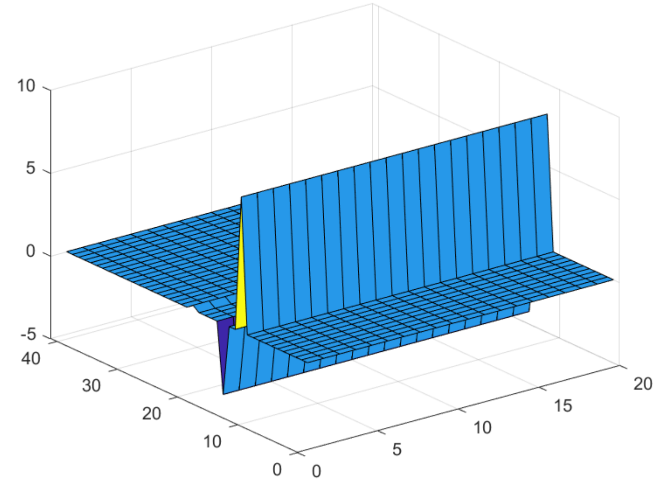

Figure A5: The plot shows the values of the function $u(i, j, t)$ on the grid $[0, L_1] \times [0, L_2]$ with the space step 1 for $t = 150$.

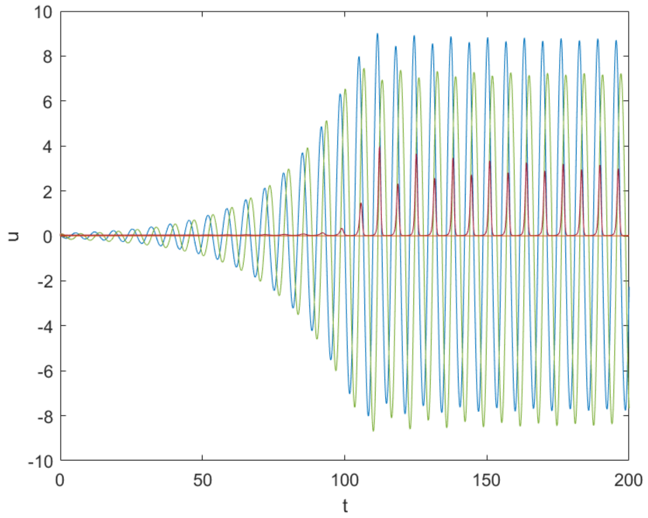

Figure A6: The plot shows how the concentrations $u(i, j, t)$ depend on time, where $t \in [0, 200]$.

where $G(x, y|\tilde{x}, \tilde{y})$ is the Green function for the operator $D\Delta - a_0^2$ under boundary conditions (A51). The choice of f_i is simple now. Let $1 < i_1 < i_2 < i_3 < L_1$ be three integers, which will be positions of layers where the reagent u is concentrated. We set

$$f(i) = -\bar{f}, i \neq i_1, i_2, i_3, \quad f(i_k) = -\lambda_k, \tag{A65}$$

where $\bar{f}, \lambda_k$ are positive and $\lambda_k << \bar{f}$. For h_i we set

$$h_i = 0, \; i \neq i_3, \; h_{i_3} = b > 0,$$

For large times t the solution u has the form

$$|u(i, j, t)| << 1, \quad i \neq i_1, i_2, i_3,$$

$$u(i_k, j, t) = X_k(t), \; k = 1, 2, 3.$$

In fact, all $u(i, j, t)$ with $i \neq i_k$ are small due to the choice of f_i. The quantities $u(i_k, j, t)$ are weakly depend on j if d is large enough. Then for X_k we have a system of differential equations with quadratic nonlinearities like to the Rössler system (under an appropriate choice of g, λ and b).

Comment. The following parameters play a key role in estimating the attractor number: the length of the system L, diffusion rate D for a fast component, ϵ^2 diffusion rate for a slow component, and a_0^2 - degradation rate for a slow component. The distance $r_K = D^{1/2}/a_0$ is a measure of the distance traveled by a molecule during its lifetime. It is sometimes referred to as the

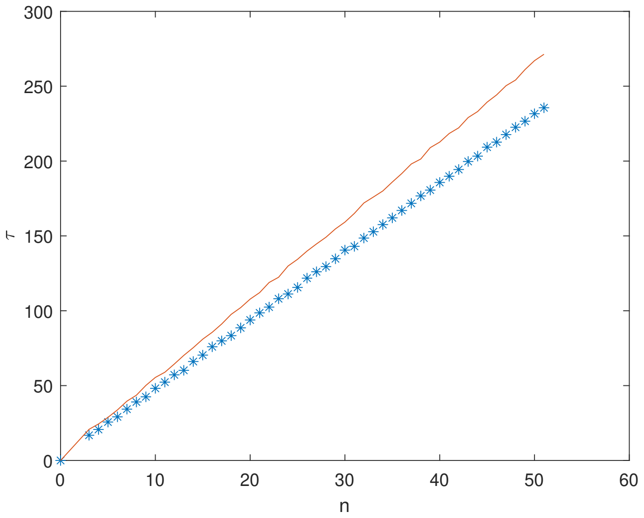

Figure A7: This image shows the dependence of the average morphogenesis time τ on the number of possible states n. This dependence has the form $E\tau \approx C(\epsilon)n$, where the constant is weakly dependent on ϵ. The upper right line corresponds to the case $\epsilon = 0.005$ and the low star line is constructed for the case $\epsilon = 0.01$. Both curves are obtained by Monte-Carlo simulations over $N_{tr} = 500$ trials. For each trial, we construct a random stochastic matrix with an absorbing state and choose a random starting state. Then we find τ and after that, we take the average over all trials.

Kuramoto length. The number of possible attractors can be roughly estimated as $\exp(L/r_K)$ if $\epsilon^2 << D$. How to explain it?

The complicated dynamics are generated by non-local interaction of closely located u-layers via fast diffusing component (it is sufficient to have 3 layers according to an example from the newly added subsection). The groups of layers should be separated to provide their almost independent functioning. If for dynamics in each group, we have two different local attractors, then if we have M groups thus the full number of attractors is 2^M. So, we have exponential dependence. The groups are well separated if distances between groups are more than the Kuramoto length. It leads to the formula $\exp(L/r_K)$. This number is an estimate from below but it is hard to believe that it may be improved. Note that exponential estimates of attractor numbers can be obtained for other models, such as the NK model.

In real conditions, the diffusion rate is bounded. Therefore, to provide adaptivity, the system should increase its size L.

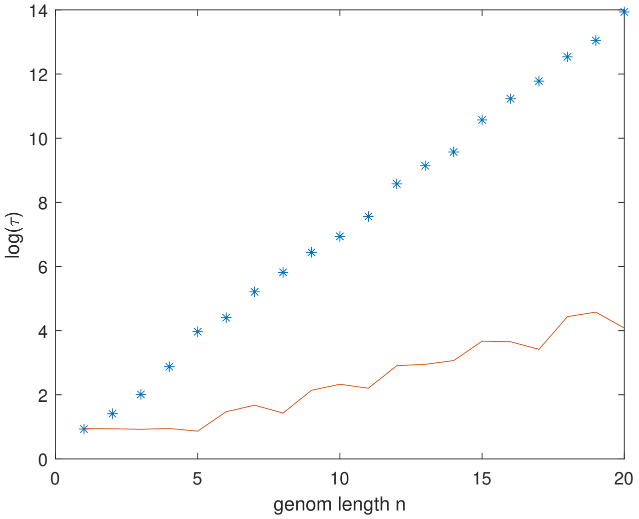

Figure A8: This image shows the dependence of the average morphogenesis time τ for the random search of a target pattern on N-dimensional hypercube. The top right star line gives values of $\log(\tau)$ for the case when the search goes in a single stage. We see that the logarithm of τ is approximately $N/2$ that corresponds to the average Hamming distance between two random bit stings of the length N. The low line describes the case when we decompose the search into three stages. Both curves are obtained by Monte-Carlo simulations over $N_{tr} = 50$ trials. For each trial, we proceed random walk on a hypercube choosing a random initial state and a random target state. For each trial we find τ and after we take the average over all trials.

1.10 Some numerical results for the system (4)

We denote $\mathbf{W}$ the $n \times n$ transition matrix with the entries $w(s, s')$ and by p the vector of state probabilities. Then iterations (**4**) satisfy the matrix equation $p(t + \Delta t) = \mathbf{W}(\xi(t))p(t)$. If ξ is absent and the transition probabilities are constants then the mean absorption time τ_s to reach the absorbing state from initial state s can be found as a positive solution of the linear algebraic system

$$\tau_s = 1 + \sum_{s' \neq s^*} w(s, s')\tau_{s'}, \quad s \neq s^*. \tag{A66}$$

In the general case of the entries, w depending on ξ we can obtain estimate $\tau_s > \bar{\tau}_s$, where $\bar{\tau}_s$ are absorption times for the Markov chain with the constant transition probabilities defined by the relations $\bar{w}(s, s') = \min_\xi w(s, s', \xi)$ for $s, s' \neq s^*$ and $\bar{w}(s^*, s) = 1 - \sum_{s'} \bar{w}(s^*, s')$.

We simulate this Markov evolution process by taking $\mathbf{W}$ with random entries. Results are represented in Figs. A7 and A9.

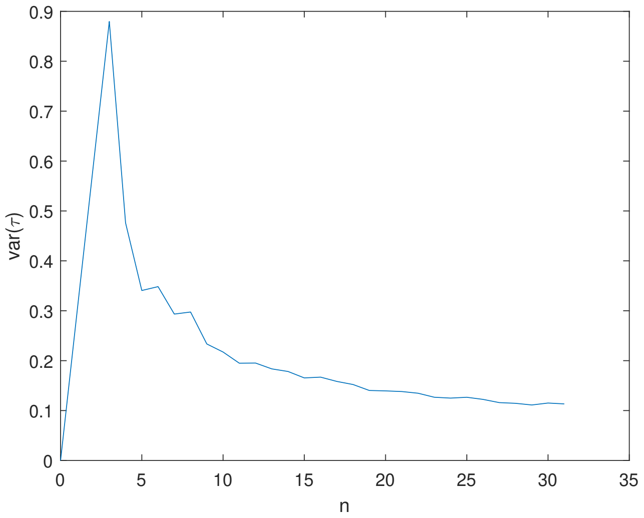

Figure A9: This plot shows the standard deviation of the morphogenesis time τ divided on the average $E\tau$. This quantity shows the accuracy of morphogenesis timing.

References

[1] S. Vakulenko, Complex attractors and patterns in reaction–diffusion systems, Journ. Dyn. Diff. Equat. 30 (2018) 175–207.

[2] G. Nicolis, I. Prigogine, Self-Organization in Nonequilibrium Systems: From Dissipative Structures to Order through Fluctuations, J. Wiley and Sons, New York, London, Sydney, Toronto, 1977.

[3] T. Tao, On the universality of the incompressible euler equations on compact manifolds, Discrete and Continuous dynamical systems 38 (3) (2018).

[4] D. Ruelle, C. Dewitt-Morette, Elements of differentiable dynamics and bifurcation theory, Physics Today (1990).

[5] D. Henry, Geometric theory of semilinear parabolic equations, Vol. 840, Springer-Verlag, Berlin, 1981.

[6] S. Vakulenko, D. Grigoriev, A. Weber, Reduction methods and chaos for quadratic systems of differential equations, Studies in Applied Mathematics 135 (2015) 225–247.

[7] M. Hirsch, H. Smith, Monotone dynamical systems, in: A. Canada, P. Drabek, A. Fonda (Eds.), Handbook of Differential Equations, Ordinary Differential Equations, second volume, Elsevier, Amsterdam, 2005.

[8] P. Constantin, C. Foias, V. Nicolaenko, R. Temam, Integral Manifolds and Inertial Manifolds for Dissipative Partial Differential Equations, Springer-Verlag, Applies Mathematical Sciences Series 70 (1988).

[9] L. Wolpert, C. Tickle, T. Jessell, Principles of development, Oxford University Press, 2002.

[10] A. Turing, The chemical basis of morphogenesis, Phil. Trans. Roy. Soc. B 237 (1952) 37–72.

[11] A. Katok, B. Hasselblatt, Introduction to the modern theory of dynamical systems, Cambridge University Press, 1997.

[12] C. Moore, Unpredactibility and undecidability in dynamical systems, Phys. Rev. Lett 64 (1990) 2354– 2357.

[13] C. Moore, Generalized shifts: Unpredactibility and undecidability in dynamical systems, Nonlinearity 4 (1991) 199–230.

[14] S. A. Vakulenko, D. Grigoriev, New way for cell differentiation: Reaction, diffusion and chaotic waves, Biosystems 212 (2022) 104605.

[15] R. J. Metzger, Sinai–ruelle–bowen measures for contracting lorenz maps and flows, Annales de l'Institut Henri Poincaré 17 (2000) 247–276.

[16] L. S. Young, What are srb measures, and which dynamical systems have them?, Journal of Statistical Physics 108 (2002) 733–754.

[17] V. I. Arnold, V. S. Afrajmovich, Y. S. Il'yashenko, L. P. Shil'nikov, Dynamical systems V, Bifurcation Theory and Catastrophe Theory, Encyclopedia of Mathematics, Springer Verlag, 1994.

[18] P. Poláčik, Complicated dynamics in scalar semilinear parabolic equations in higher space dimension, Journal of Differential Equations 89 (2) (1991) 244 – 271.

[19] P. Poláčik, High-dimensional ω-limit sets and chaos in scalar parabolic equations, Journal of Differential Equations 119 (1) (1995) 24 – 53.

[20] S. Vakulenko, Dissipative systems generating any structurally stable chaos, Advances in Differential Equations 5 (7-9) (2000) 1139–1178.

[21] S. Vakulenko, I. Morozov, O. Radulescu, Maximal switchability of centralized networks, Nonlinearity 29 (8) (2016) 2327.

[22] S. Vakulenko, Strange attractors for oberbeck–boussinesq model, Journ. Dyn. Diff. Equat. 33 (2021) 303–343.

[23] S. Smale, Differentiable dynamical systems, Bull. Amer. Math. Soc. 73 (6) (1967) 747–817.

[24] P. Constantin, Integral manifolds and inertial manifolds for dissipative partial differential equations, Vol. 70, Springer Science & Business Media, 1989.

[25] J. Carr, R. Pego, Metastable patterns, Comm. on Pure and Applied Math. 42 (1989) 523–576.

[26] H. Meinhardt, Turing's theory of morphogenesis of 1952 and the subsequent discovery of the crucial role of local self-enhancement and long-range inhibition, Interface Focus 2 (2012) 407–416.
